# Supplementary material for: Vegetation type is an important predictor of the arctic summer land surface energy budget
Source: Nat Commun. 2022 Oct 31;13:6379. doi: 10.1038/s41467-022-34049-3 (PMC9622844; doi:10.1038/s41467-022-34049-3)
Supplement: Supplementary file 1 — Supplementary Information [file 41467_2022_34049_MOESM1_ESM.pdf]

Supplementary Information

Vegetation Type is an Important Predictor of the Arctic Summer Land Surface Energy Budget

Jacqueline Oehri[1,2,#*], Gabriela Schaepman-Strub[1,#*], Jin-Soo Kim[1,3,†], Raleigh Grysko[1,†], Heather Kropp[4,†], Inge Grünberg[5,†], Vitalii Zemlianskii[1,†], Oliver Sonnentag[6,†], Eugénie S. Euskirchen[7,†], Merin Reji Chacko[1,8,9,†], Giovanni Muscari[10], Peter D. Blanken[11], Joshua F. Dean[12], Alcide di Sarra[13], Richard J. Harding[14], Ireneusz Sobota[15], Lars Kutzbach[16], Elena Plekhanova[1], Aku Riihelä[17], Julia Boike[5,18], Nathaniel B. Miller[19], Jason Beringer[20], Efrén López-Blanco[21,22], Paul C. Stoy[19], Ryan C. Sullivan[23], Marek Kejna[24], Frans-Jan W. Parmentier[25,26], John A. Gamon[27], Mikhail Mastepanov[22,28], Christian Wille[29], Marcin Jackowicz-Korczynski[22,26], Dirk N. Karger[30], William L. Quinton[31], Jaakko Putkonen[32], Dirk van As[33], Torben R. Christensen[22,28], Maria Z. Hakuba[34], Robert S. Stone[35], Stefan Metzger[36,37], Baptiste Vandecrux[33], Gerald V. Frost[38], Martin Wild[39], Birger Hansen[40], Daniela Meloni[41], Florent Domine[42,43], Mariska te Beest[44,45], Torsten Sachs[29], Aram Kalhori[29], Adrian V. Rocha[46], Scott N. Williamson[47], Sara Morris[48], Adam L. Atchley[49], Richard Essery[50], Benjamin R. K. Runkle[51], David Holl[16], Laura D. Riihimaki[52,53], Hiroki Iwata[54], Edward A. G. Schuur[55], Christopher J. Cox[48], Andrey A. Grachev[56], Joseph P. McFadden[57], Robert S. Fausto[33], Mathias Göckede[58], Masahito Ueyama[59], Norbert Pirk[60], Gijs de Boer[48,52,61], M. Syndonia Bret-Harte[7], Matti Leppäranta[62], Konrad Steffen[30,§], Thomas Friborg[40], Atsumu Ohmura[39], Colin W. Edgar[7], Johan Olofsson[63], Scott D. Chambers[64]

*Corresponding authors: Jacqueline Oehri, jacqueline.oehri@gmail.com & Gabriela Schaepman-Strub, gabriela.schaepman@ieu.uzh.ch
#These authors jointly supervised this work. †These authors contributed equally.
§Deceased 8 August 2020

1. Department of Evolutionary Biology and Environmental Studies, University of Zurich, Winterthurerstrasse 190, 8057 Zurich, Switzerland
2. Department of Biology, McGill University, 1205 Docteur Penfield, H3A 1B1 Montreal, Quebec, Canada
3. Low-Carbon and Climate Impact Research Centre, School of Energy and Environment, City University of Hong Kong, Tat Chee Ave, Kowloon Tong, Hongkong, People's Republic of China
4. Environmental Studies Program, Hamilton College, 198 College Hill Rd, Clinton, NY, USA
5. Permafrost Research Section, Alfred-Wegener Institute, Telegrafenberg, 14473, Potsdam, Germany
6. Département de géographie, Université de Montréal, 2900 Edouard Montpetit Blvd, Montreal, Quebec H3T 1J4, Canada
7. Institute of Arctic Biology, University of Alaska Fairbanks, 2140 Koyukuk Dr, Fairbanks, AK, USA
8. Institute of Terrestrial Ecosystems, ETH Zurich, CHN, Universitätstrasse 16, 8006 Zurich, Switzerland

9. Land Change Science Unit, Swiss Federal Institute for Forest, Snow and Landscape Research (WSL), Zürcherstrasse 111, 8903 Birmensdorf ZH, Switzerland
10. Istituto Nazionale di Geofisica e Vulcanologia, Via di Vigna Murata, 605, Rome, Italy
11. Department of Geography, University of Colorado, Boulder, USA
12. School of Geographical Sciences, University of Bristol, School of Geographical Sciences, University Rd, Bristol, United Kingdom
13. Department for Sustainability, ENEA, Via Enrico Fermi 45, Frascati, Italy
14. UK Centre for Ecology & Hydrology (UKCEH), MacLean Bldg, Benson Ln, Crowmarsh Gifford, Wallingford, UK
15. Department of Hydrology and Water Management, Faculty of Earth Sciences and Spatial Management, Nicolaus Copernicus University, Lwowska, 87-100 Toruń, Poland
16. Center for Earth System Research and Sustainability (CEN), University of Hamburg, Bundesstrasse 53, 20146 Hamburg, Germany
17. Finnish Meteorological Institute, Erik Palménin aukio 1, 00560 Helsinki, Finland
18. Geography Department, Humboldt-Universität zu Berlin, Unter den Linden 6, 10117 Berlin, Germany
19. University of Wisconsin-Madison, Madison, WI, USA
20. School of Agriculture and Environment, The University of Western Australia, 35 Stirling Hwy, Crawley WA 6009, Australia
21. Department of Environment and Minerals, Greenland Institute of Natural Resources, Kivioq 2, Nuuk 3900, Greenland
22. Department of Ecoscience, Aarhus University, Nordre Ringgade 1, 8000 Aarhus C, Denmark
23. Environmental Science Division, Argonne National Laboratory, 9700 S Cass Ave, Lemont, IL, USA
24. Department of Meteorology and Climatology, Faculty of Earth Sciences and Spatial Management, Nicolaus Copernicus University, Lwowska, 87-100 Toruń, Poland
25. Center for Biogeochemistry of the Anthropocene, Department of Geosciences, University of Oslo, Sem Sælands vei 1, 0371 Oslo, Norway
26. Department of Physical Geography and Ecosystem Science, Lund University, Geocentrum II, Sölvegatan 12, 223 62 Lund, Sweden
27. University of Nebraska - Lincoln, 1400 R St, Lincoln, NE, USA
28. Oulanka Research Station, University of Oulu, Pentti Kaiteran katu 1, 90570 Oulu, Finland
29. GFZ German Research Centre for Geosciences, Wissenschaftspark Albert Einstein, Telegrafenberg, 14473 Potsdam, Germany
30. Swiss Federal Institute for Forest, Snow, and Landscape Research (WSL), Zürcherstrasse 111, 8903 Birmensdorf ZH, Switzerland
31. Cold Regions Research Centre, Wilfrid Laurier University, 75 University Ave W, Waterloo, ON, Canada
32. Harold Hamm School of Geology and Geological Engineering, University of North Dakota, Grand Forks, ND, USA
33. Department of Glaciology and Climate, Geological Survey of Denmark and Greenland (GEUS), Øster Voldgade 10, 1350 Copenhagen, Denmark
34. Jet Propulsion Laboratory, CalTech, 4800 Oak Grove Dr, Pasadena, CA, USA
35. NOAA Global Monitoring Laboratory, 325 Broadway, Boulder, CO, USA, affiliate (retired)
36. National Ecological Observatory Network, Battelle, 1685 38th St #100, Boulder, CO, USA
37. Dept. of Atmospheric and Oceanic Sciences, University of Wisconsin-Madison, 1225 W Dayton St, Madison, WI, USA
38. Alaska Biological Research, Inc., 2842 Goldstream Rd, Fairbanks, AK, USA

39. Institute for Atmospheric and Climate Science, ETH Zurich, CHN, Universitätstrasse 16, 8006 Zurich, Switzerland
40. Department of Geosciences and Natural Resource Management, University of Copenhagen, Rolighedsvej 23, 1958 Frederiksberg, Denmark
41. Department for Sustainability, ENEA, Lungotevere Grande Ammiraglio Thaon di Revel, 76, Rome, Italy
42. Department of Chemistry, Université Laval, Pavillon Alexandre-Vachon, 1045 Av. de la Médecine, G1V 0A6 Québec, QC, Canada
43. Takuvik Laboratory, CNRS-INSU, Département de Biologie, Université Laval, Pavillon Alexandre-Vachon, 1045 Av. de la Médecine, G1V 0A6 Québec, QC, Canada
44. Copernicus Institute of Sustainable Development, Utrecht University, Vening Meinesz building, Princetonlaan 8a, 3584 CB Utrecht, The Netherlands
45. Centre for African Conservation Ecology, Nelson Mandela University, University Way, Summerstrand, Gqeberha, 6019, Port Elizabeth, South Africa
46. Department of Biological Sciences, University of Notre Dame, 100 Galvin Life Sciences, Notre Dame, IN, USA
47. Polar Knowledge Canada, Canadian High Arctic Research Station, 1 rue Uvajuq place, CP 2150, Cambridge Bay, NU, Canada
48. NOAA Physical Sciences Laboratory, 325 Broadway, Boulder, CO, USA
49. Los Alamos National Laboratory, Bikini Atoll Rd., SM 30, Los Alamos, NM, USA
50. School of Geosciences, University of Edinburgh, Drummond St, Edinburgh EH8 9XP, UK
51. Department of Biological & Agricultural Engineering, University of Arkansas, 1164 W Maple St, Fayetteville, AR, USA
52. CIRES (Cooperative Institute for Research in Environmental Sciences), 216 UCB, University of Colorado Boulder campus, Boulder, CO, USA
53. NOAA Global Monitoring Laboratory, 325 Broadway, Boulder, CO, USA
54. Department of Environmental Science, Shinshu University, 3 Chome-1-1 Asahi, Matsumoto, Nagano 390-8621, Japan
55. Center for Ecosystem Science and Society, Northern Arizona University, S San Francisco St, Flagstaff, AZ, USA
56. DEVCOM Army Research Laboratory, Owen Rd, White Sands Missile Range, NM, USA
57. Department of Geography and Earth Research Institute, University of California Santa Barbara, 5816 Ellison Hall, Isla Vista, CA, USA
58. Department of Biogeochemical Signals, Max Planck Institute for Biogeochemistry, Hans-Knöll-Straße 10, 07745 Jena, Germany
59. Osaka Metropolitan University, Sakai, Kita Ward, Umeda, 1 Chome−2−2-600, Osaka, Japan
60. Department of Geosciences, University of Oslo, Sem Sælands vei 1, 0371 Oslo, Norway
61. IRISS (Integrated Remote and In Situ Sensing), University of Colorado, Boulder, CO, USA
62. University of Helsinki, Yliopistonkatu 4, 00100 Helsinki, Finland
63. Department of Ecology and Environmental Science, Umeå University, Linnaeus väg 4-6, 907 36 Umeå, Sweden
64. ANSTO Lucas Heights, New Illawarra Rd, Lucas Heights NSW 2234, Australia

Supplementary Figures

Supplementary Figure 1

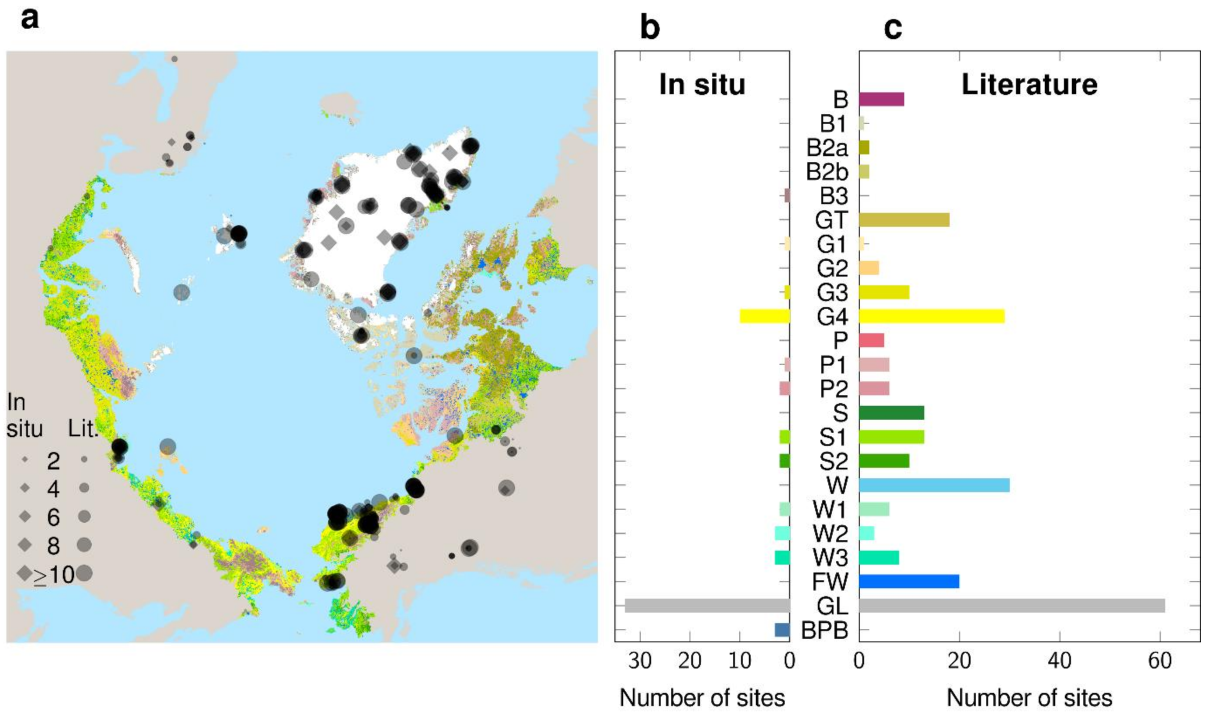

Supplementary Figure 1. **Spatio-temporal coverage of surface energy budget (SEB)-measurement sites reported in the collected literature (literature data; circles) and in situ observations (SEB-data; diamonds, a), coverage of vegetation types classified according to main plant physiognomies (b). a** We collected literature on the Web of Science Core Collection and found n=358 (252 unique) sites (circles) present in 148 studies investigating surface energy fluxes (Supplementary Table 1) on vegetation or glacier surfaces above 60° N. Estimates of surface energy fluxes derived from local in situ measurements were collected from the open-access data repositories Ameriflux, FLUXNET, GC-Net, PROMICE, ICOS, GEM and AON (diamonds; Supplementary Table 3). These networks harbor data for 64 sites on vegetation or glacier surfaces above 60° N. Size of dots: study duration. Map coloring corresponds to the raster circumpolar Arctic vegetation map (CAVM[19]) types: B1-4: barrens; GT and G1-4: graminoid; P1-2: prostrate-shrub; S1-2: erect-shrub; W1-3: wetlands; GL: glacier; FW: freshwater. We extracted the in situ vegetation type ('Vegetation type') from site descriptions for each study site in the in situ SEB-data (**b**) and literature data (**c**) and counted the number of sites per vegetation type. Vegetation types correspond to CAVM types (in some cases only resolvable to the main plant physiognomy without number) plus one additional class: BPB (boreal peat bog). See Supplementary Figure 2 for maps where study site locations are mapped for each vegetation type separately. Source data are provided as a Source Data file.

Supplementary Figure 2

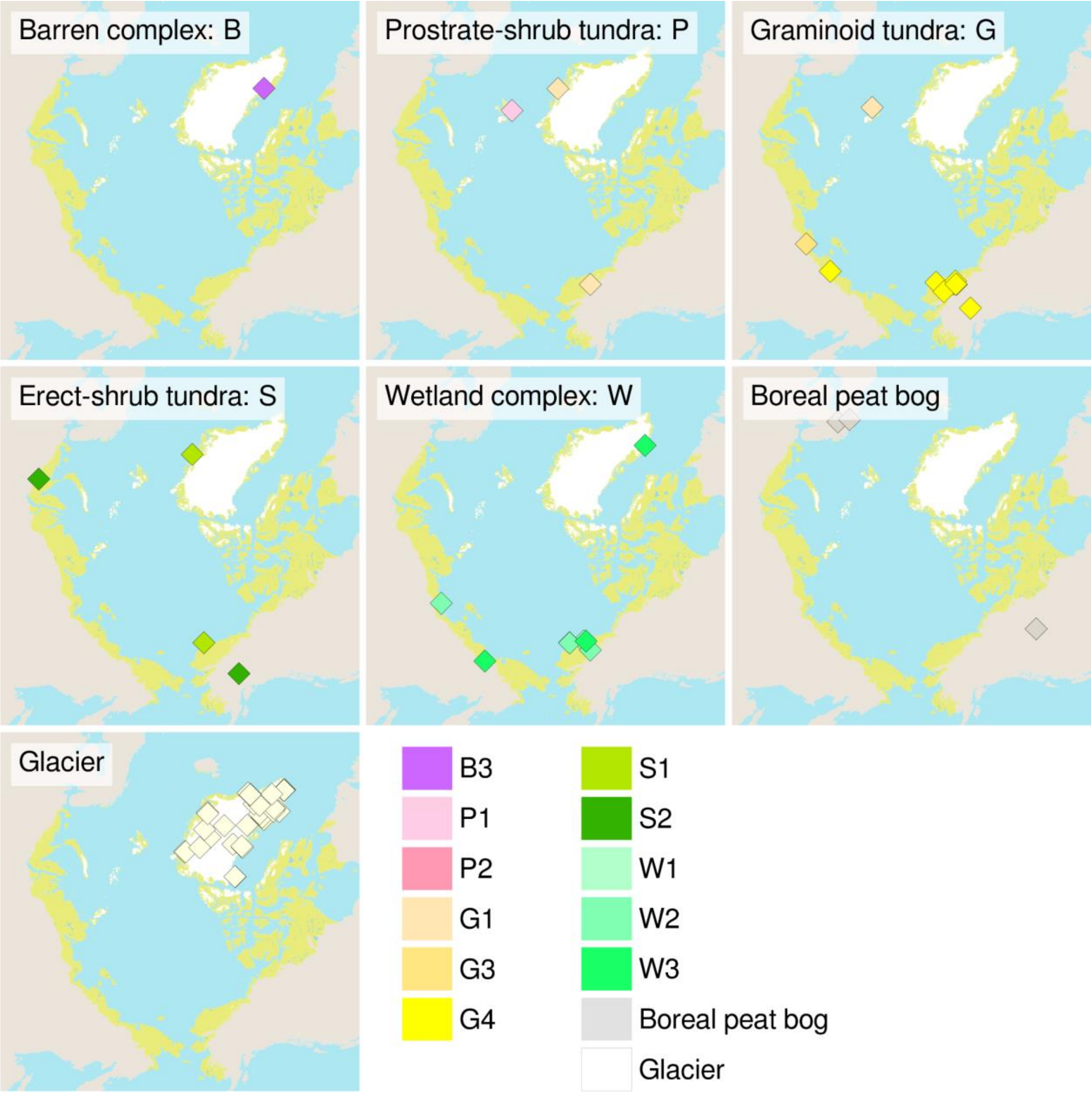

Supplementary Figure 2. **Distribution of study sites in the in situ observations data (SEB-data), grouped by the Vegetation type variable.** Detailed Vegetation types: B3: non-carbonate mountain complex; G1: graminoid, forb, cryptogam tundra; G3: non-tussock sedge, dwarf-shrub, moss tundra; G4: tussock-sedge, dwarf-shrub, moss tundra; P1: prostrate dwarf-shrub, herb, lichen tundra; P2: prostrate/hemi-prostrate dwarf-shrub, lichen tundra; S1: erect dwarf-shrub, moss tundra; S2: low-shrub, moss tundra; W1: sedge/grass, moss wetland complex; W2: sedge, moss, dwarf-shrub wetland complex; W3: sedge, moss, low-shrub wetland complex. The following detailed CAVM[19] classes are not contained in the data: B1: cryptogam, herb barren; B2a: cryptogam, barren complex; B2b: cryptogam, barren, dwarf-shrub complex; B4: carbonate mountain complex; G2: graminoid, prostrate dwarf-shrub, forb, moss tundra. See Methods and ref.[19] for more details.

Supplementary Figure 3

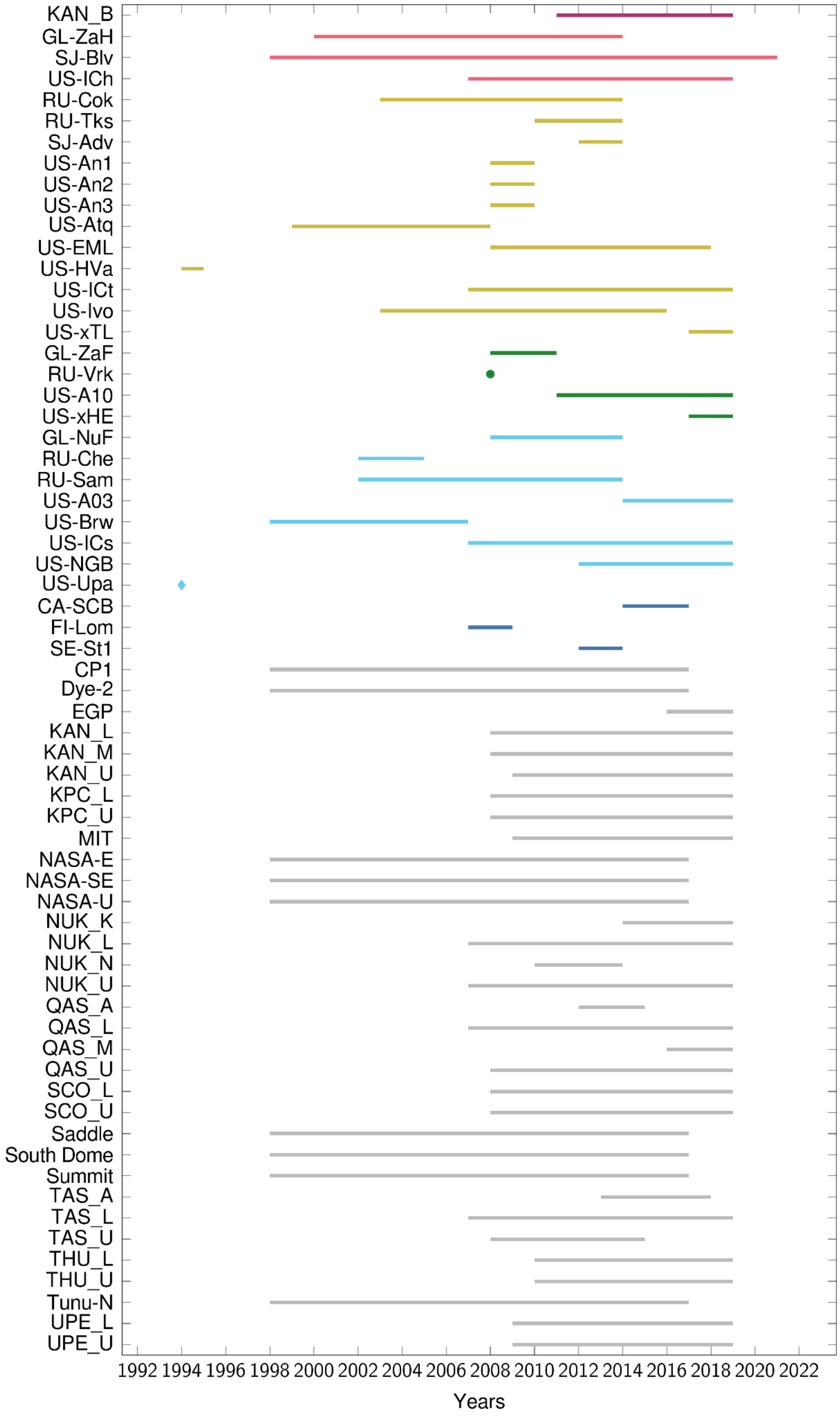

Supplementary Figure 3. **Temporal coverage of in situ observations in the SEB-data.** Shown are respective times when data of either net radiation (R_{net}), sensible heat flux (H) or latent heat flux (LE) is available at a specific study site. Sites are grouped according to Vegetation type; colors correspond to barren complex (purple); prostrate-shrub tundra (rose); graminoid tundra (yellow green); erect-shrub tundra (dark green); wetlands (light blue); boreal peat bog (dark blue) and glacier (grey). In total, there are 64 sites covering the time 1994-2021; the average nr. of years covered by a site are 10 ± 5.87. Source data are provided as a Source Data file.

Supplementary Figure 4

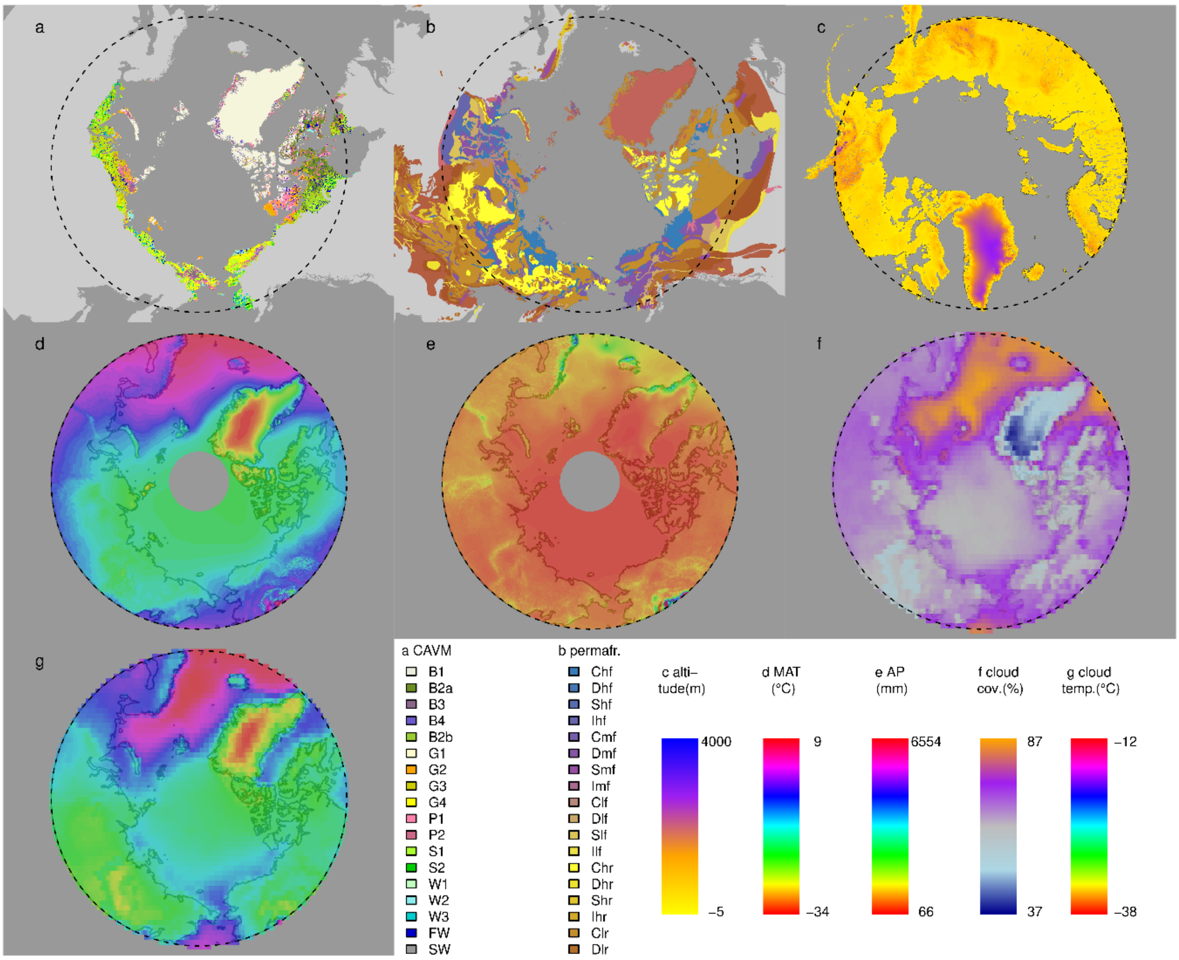

Supplementary Figure 4. **Maps of ancillary geographic data used to derive surface energy budget (SEB)-drivers. a** CAVM raster map[19] with the CAVM types: B1-4: barrens; G1-4: graminoid; P1-2: prostrate-shrub; S1-2: erect-shrub; W1-3: wetlands; GL: glacier; FW: freshwater; SW: seawater, NAr: non-Arctic vegetation. **b** permafrost map[61], with information on permafrost extent (C: continuous, D: discontinuous, S: sporadic, I: isolated patches) and ground ice content (h: high, m: medium, l: low). **c** altitude (m above sea level): ArcticDEM mosaic product: arcticdem_mosaic_100m_v3.0[62]. **d** mean annual air temperature (°C): CHELSA V2.1 submodel CMIP5 "bio10_01" variable[53,54]. **e** annual precipitation (mm): CHELSA V2.1 submodel CMIP5 "bio10_12" variable[55]. **f** cloud cover (%): "cldamt" product in ISCCP-Basic-H series: ISCCP-Basic.HGM.v01r00.GLOBAL[59,60]. **g** cloud-top temperature (°C) : "tc" product in ISCCP-Basic-H series: ISCCP-Basic.HGM.v01r00.GLOBAL[59,60]. The dashed line corresponds to the 60° N latitude. A list of data sources used in this study is contained in Supplementary Table 3.

Supplementary Figure 5

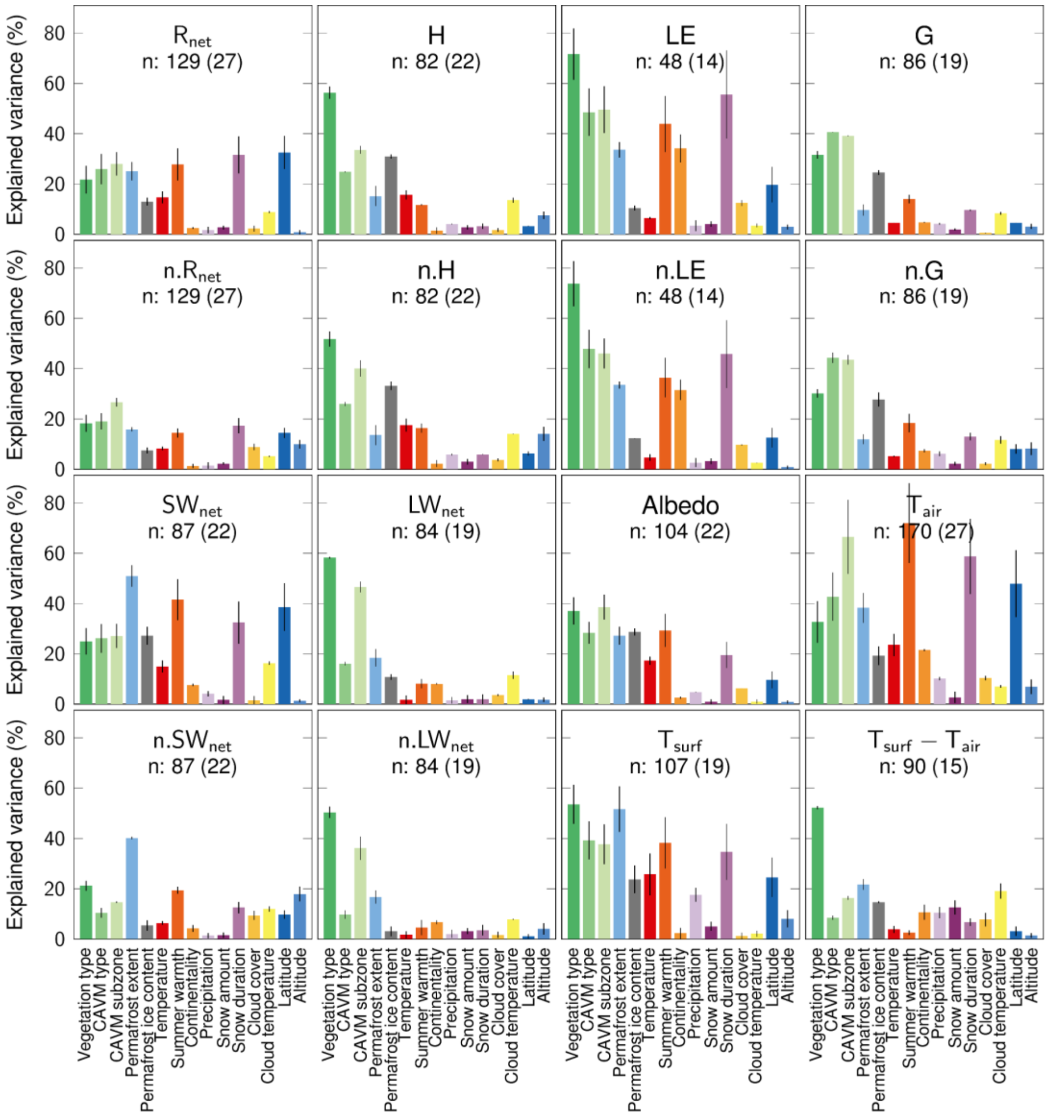

Supplementary Figure 5. **Relative importance of 15 drivers of the surface energy budget (SEB) for average summer magnitudes of fluxes and components of the SEB at non-glacier sites.** Bars show the mean (bar height) and range (lines for each bar) of explained variance (%) for each SEB-driver and corresponding SEB-flux or component averaged across all possible models with 2 predictors in the variance partitioning analysis (Methods). SEB-fluxes and components (Wm^{-2}): R_{net}: net radiation, H: sensible heat flux, LE: latent heat flux, G: ground heat flux. $S(L)W_{net}$: net shortwave (longwave) radiation. Surface energy fluxes with “n.”-prefix: normalized fluxes in % of maximum potential incoming shortwave radiation, Albedo: albedo; T_{air}: air temperature in °C; T_{surf}: surface temperature in °C; T_{surf}-T_{air}: difference between T_{surf} and T_{air} in °C. SEB-drivers: Vegetation type (dark green): local-scale, in situ vegetation type; CAVM type (green): landscape-scale, dominant vegetation type (surrounding area with radius of 500m); CAVM subzone (light green): bioclimatic subzone; Permafrost extent (light blue): permafrost spatial extent; Permafrost ice content (grey): permafrost ground ice content; Temperature (red): mean annual air temperature; Summer warmth (dark orange): summer warmth index; Continentality (orange): Conrad’s continentality index; Precipitation (light purple): mean annual precipitation; Snow amount (dark purple): mean annual snow water equivalent; Snow duration (purple): median annual snow cover duration; Cloud cover (light orange): mean cloud cover; Cloud temperature (yellow): mean cloud-top temperature; Latitude (dark blue): latitude (WGS84); Altitude (light blue): mean altitude (surrounding area with radius of 500 m; see Methods and Supplementary Table 1). n: average nr. of site years with average nr. of sites in parentheses. Results are based on the vegetation subset of our data (excluding glacier sites): nr. of sites: 31, nr. of site years: 234, period: 1994-2021. Source data are provided as a Source Data file.

Supplementary Figure 6

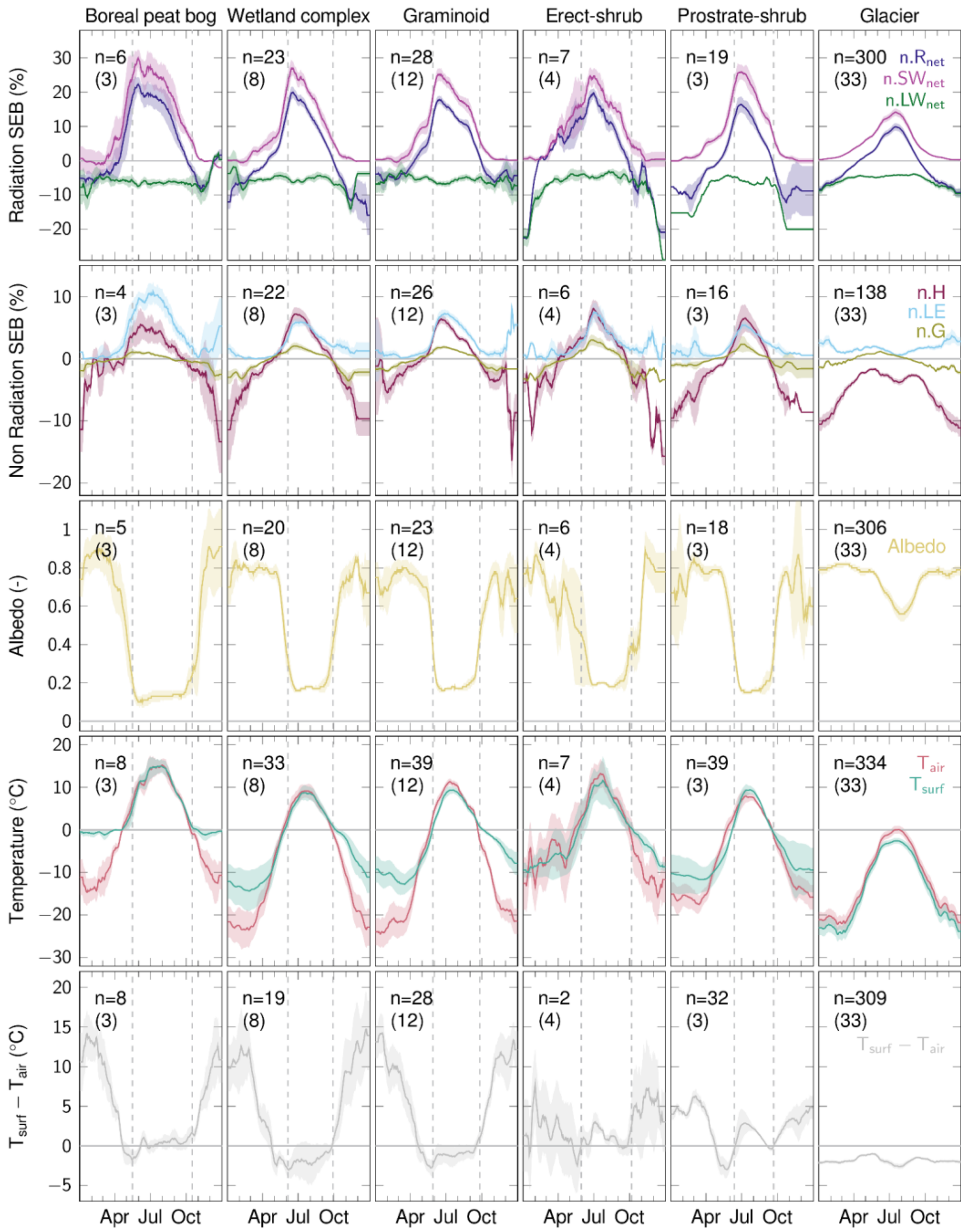

Supplementary Figure 6. **Seasonalities for selected fluxes and components of the surface energy budget (SEB).** SEB-flux and component values were averaged for each study site for each day of year (DOY) across all years available and then averaged (mean ± s.e.) for each DOY and smoothed (15-day moving average) for each Vegetation type. Average number of site years (n) and number of study sites (in parentheses) across all DOYs and SEB fluxes/components are indicated in the top left corner of each inset figure. The area within the vertical gray lines represents the median snow free period across the years 2000-2020 (MODIS[38]; Methods), averaged for each Vegetation type (boreal peat bog, wetland complex, graminoid tundra, erect-shrub tundra, prostrate-shrub tundra, glacier). Normalized surface energy fluxes in percent of maximum potential incoming shortwave radiation: n.R_{net} (dark blue): net radiation; n.SW_{net} (purple): net shortwave radiation; n.LW_{net} (green): net longwave radiation; n.H (dark red): sensible heat flux; n.LE (light blue): latent heat flux; n.G (yellow green): ground heat flux; Albedo (yellow): albedo; T_{surf} (mint): surface temperature and T_{air} (red): air temperature in °C; T_{surf}-T_{air} (grey): difference between surface and air temperature in °C (Supplementary Table 1). Results are based on the data subset of the period 2000-2021 and excluding barren vegetation type because of missing R_{net} data: nr. sites=61, nr. site years=617. Source data are provided as a Source Data file.

Supplementary Figure 7

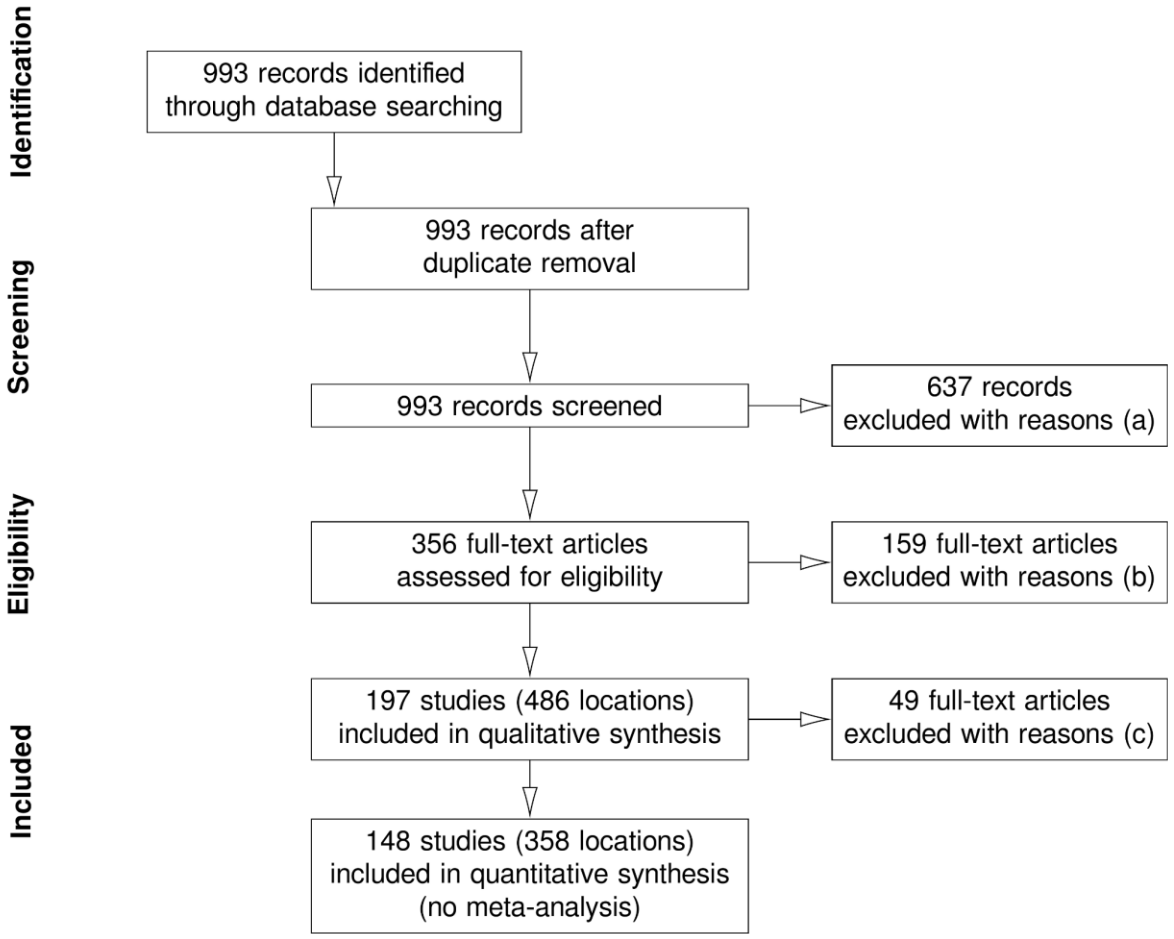

Supplementary Figure 7**. PRISMA flow diagram (adapted from the downloadable template[1]), depicting the selection procedure of collected literature for our systematic review.** Reasons to exclude studies: **a** studies that i. did not cover terrestrial land areas (e.g. studies of marine and ocean surfaces, or studies of extra-terrestrial energy fluxes), ii. were conceptual studies without measured data, iii. did not report on any of the surface energy fluxes and components we deemed as essential (Supplementary Table 1), iv. were not peer-reviewed research articles, v. were not primary research articles (e.g. meta-analyses and reviews), vi. did only cover times before 1950. **b** studies that i. did not contain any estimates for specific geographic locations at local spatial scale (<10 km^2 area), ii. did not contain in situ measurements of essential surface energy fluxes and components (Supplementary Table 1), iii. covered only locations below 50° N (e.g. alpine tundra only). **c** Studies that i. covered only locations below 60° N, ii. did not cover surface energy flux measurements on vegetation or glacier ice (i.e. studies on forest, forest ecotone, lake, urban or crop land-cover). These eligibility criteria were developed according to the PICOS approach as follows: 1. Participants: studies reporting on essential surface energy fluxes and components (Supplementary Table 1), across the pan-Arctic and subarctic regions > 60° N, across all available years >1950; 2. Interventions & Comparators: studies reporting on SEB-drivers of interest: climate (including mean annual air temperature and annual precipitation), vegetation type, permafrost type, topography, snow characteristics and seasonality, cloud properties, and time of year. 3. Outcomes: magnitude and timing of surface energy fluxes, identity of surface energy fluxes and components, identity of SEB-drivers, latitude, longitude, land-cover type of study locations, seasons assessed, spatio-temporal extent of assessed surface energy fluxes and components and -drivers, data and methods used for inference of surface energy fluxes/components and drivers (i.e. modeled, directly measured, interpolated, remotely sensed, etc.), surface energy budget closure assumption; 4. Study design: comparative, experimental and modeling studies containing in situ measurements of surface energy fluxes and components[1].

Supplementary Figure 8

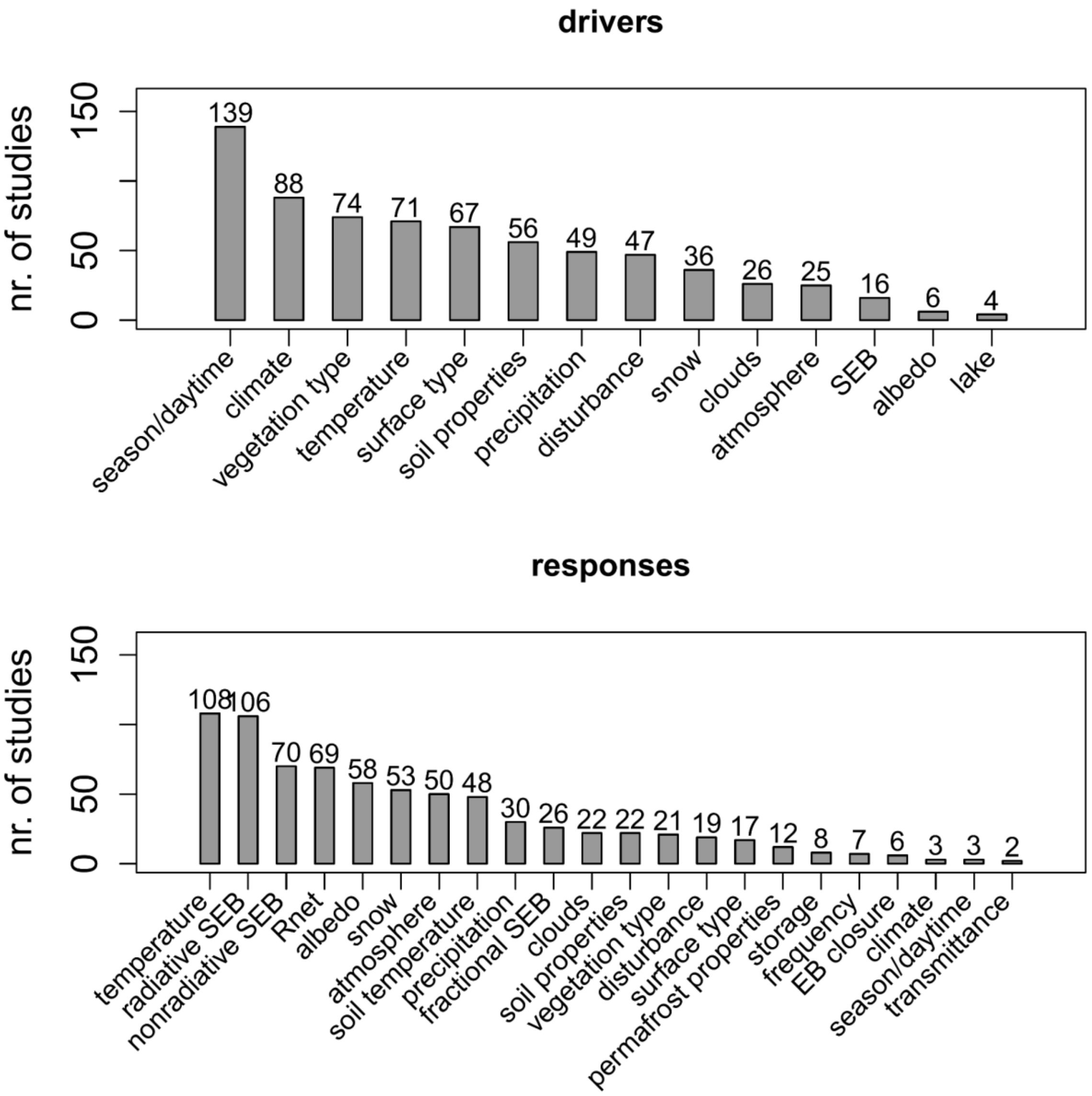

Supplementary Figure 8. **Drivers (top panel) and responses (bottom panel) assessed in the collected literature on Arctic or high-latitude surface energy budgets (SEB, n=148 studies on vegetation or glaciers above 60°N).** Drivers: variables considered as driving factors in studies, typically on x-axes in figures; responses: variables considered as response variables in studies, typically on y-axes in figures. Bar height and numbers refer to the numbers of studies that contain a respective driver or response variable category. Radiative SEB contains incoming, outgoing and net radiative energy fluxes; nonradiative SEB contains convective and conductive sensible, latent and ground heat fluxes, as well as energy used for melting snow and ice; fractional SEB contains bowen ratio, evaporative fraction and energy fluxes indicated as fraction of absorbed or incoming radiation. Literature data on surface energy budgets across the Arctic was systematically searched and collected on the ISI Web of Science (Methods). We find that the majority of studies (82%) simultaneously assessed several surface energy fluxes/components and -drivers. Air temperature, radiative and non-radiative surface energy fluxes are most often assessed as response variables (bottom panel), whereas many studies assess season/daytime, climatic factors and vegetation type as drivers (top panel). An overwhelming majority of studies (139 of 148 studies, i.e. 94%), assessed time (i.e. time of year or time of day) as a driver.

Supplementary Figure 9

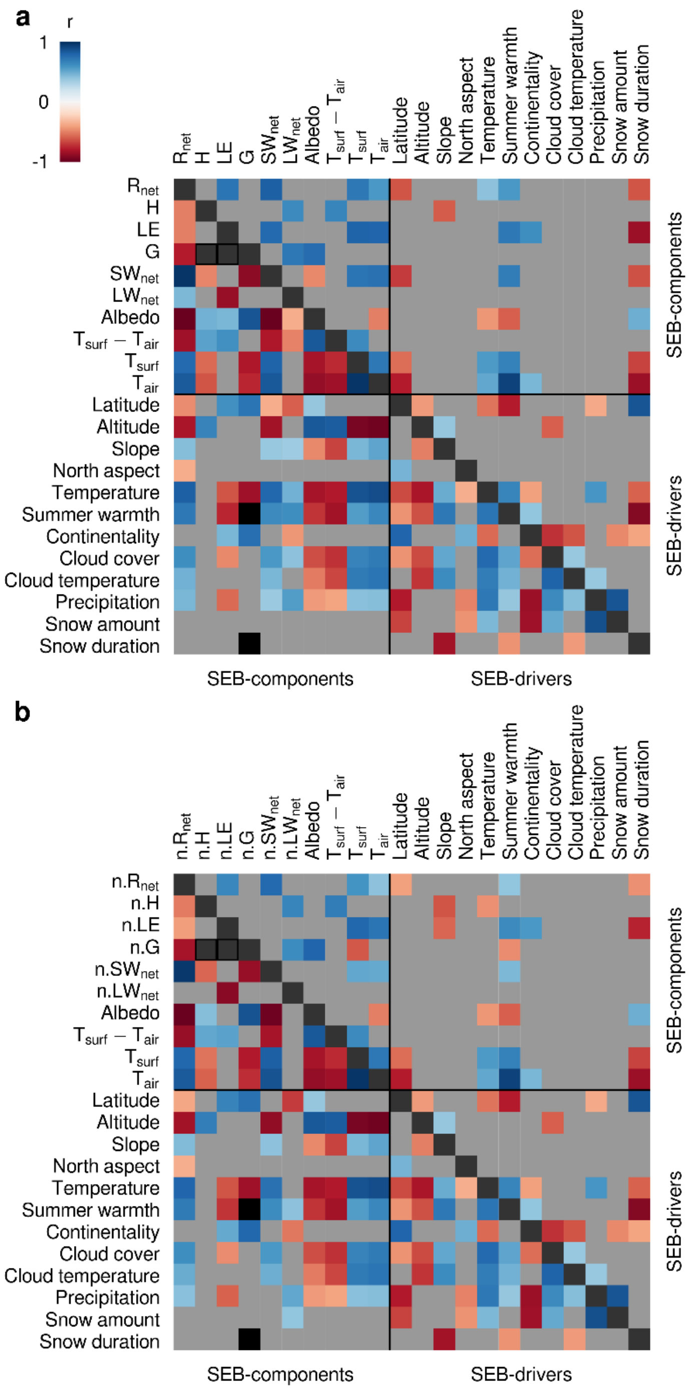

Supplementary Figure 9. Relationships among components and drivers of the summer surface energy budget (SEB) for the "vegetation" SEB-data subset (upper right triangles) and the „glacier" SEB-data subset (lower left triangles). Pearson correlation coefficients (r) for site-dependent SEB-drivers and mixed-model estimates of surface energy flux/component mean summer (JJA) magnitudes. Significant negative (red) and positive (blue) correlations (i.e. P-value<0.05) are indicated with colored rectangles. Light grey rectangles: insignificant relationships. Dark grey rectangles: correlations with the same variable or correlation with fewer than 5 sites involved. Note that flux direction convention is positive away from the surface for non-radiative fluxes. **a** analysis with mean surface energy flux values in Wm^{-2}; **b** analysis with normalized surface energy fluxes expressed in % of potential incoming shortwave radiation (prefix "n." for corresponding surface energy fluxes). R_{net}: net radiation; SW_{net}: net shortwave radiation; LW_{net}: net longwave radiation; H: sensible heat flux; LE: latent heat flux; G: ground heat flux; Albedo: albedo; T_{surf}: surface temperature; T_{air}: air temperature; T_{surf}-T_{air}: difference between surface and air temperature; SEB-drivers: Altitude, Slope and North aspect: mean in surrounding area with radius of 500m; Temperature: mean annual air temperature; Summer warmth: summer warmth index; Continentality: conrad's continentality index; Cloud cover: mean cloud cover; Cloud temperature: mean cloud-top temperature; Precipitation: mean annual precipitation; Snow amount: mean annual snow water equivalent; Snow duration: median annual snow cover duration; see Methods and Supplementary Table 1 for more details). Average nr. of site years (sites) for the 'vegetation' dataset (excluding glacier sites, upper right triangle): 127 (30) and for 'glacier' dataset (excluding vegetation sites, lower left triangle): 333 (32). The magnitude and direction of correlations differ between the vegetation and glacier datasets and significance tends to be weaker in the vegetation dataset, even though the number of study sites is comparable. In the glacier dataset, cloud and precipitation variables show strong correlations with surface energy components, in contrast to the vegetation dataset. In both datasets, Summer warmth shows strong relationships with surface energy components. Results for absolute (**a**) and normalized (**b**) surface energy fluxes are relatively similar. Source data are provided as a Source Data file.

Supplementary Tables

Supplementary Table 1

Supplementary Table 1. **List of essential surface energy fluxes and components, as well as surface energy budget (SEB)-drivers used in this study.** Main category: surface energy fluxes and components: energy fluxes, atmospheric state variables and surface properties that affect or correlate with the surface energy budget[18,19]; SEB-drivers: environmental factors that have been found to influence surface energy fluxes; detailed category: detailed categories as used in this study; variable name: abbreviations as used in this study; explanation: corresponding explanations; unit: corresponding variable unit. See Methods section for details on data processing for variable derivation.

nr.	main category	detailed category	variable name	explanation	unit
1	Surface energy flux or component	radiative SEB	R_{net}*	net radiation	Wm^{-2}
2			SW_{net}*	surface net shortwave radiation	Wm^{-2}
3			LW_{net}*	surface net longwave radiation	Wm^{-2}
4			SW_{in}*	surface incoming shortwave radiation	Wm^{-2}
5			SW_{out}*	surface outgoing shortwave radiation	Wm^{-2}
6			LW_{in}*	surface incoming longwave radiation	Wm^{-2}
7			LW_{out}*	surface outgoing longwave radiation	Wm^{-2}
8		non-radiative SEB	H*	sensible heat flux	Wm^{-2}
9			LE*	latent heat flux	Wm^{-2}
10			G*	ground heat flux	Wm^{-2}
11		albedo	albedo	albedo	no unit
12		temperature	T_{surf}	surface temperature	°C
13			T_{air}	air temperature	°C
14			T_{surf}-T_{air}	gradient; i.e. difference between surface and air temperature	°C
15	SEB-driver	vegetation	Vegetation type	local-scale, in situ vegetation type	factor with 7 levels
16			CAVM type	landscape-scale dominant Arctic vegetation type	factor with 7 levels
17		climate	CAVM subzone	bioclimatic subzone	factor with 6 levels
18			Continentality	Conrad's continentality index	°C
19			Summer warmth	summer warmth index	°C
20			Temperature	mean annual air temperature	°C
21			Precipitation	annual precipitation	mm
22			Snow amount	annual snowfall (precipitation when air temperature <=0°C)	mm
23		clouds	Cloud cover	cloud cover	%
24			Cloud temperature	cloud top temperature	°C
25		permafrost	Permafrost extent	permafrost extent	factor with 5 levels
26			Permafrost ice content	permafrost ground ice content	factor with 5 levels
27		topography	Altitude	altitude	m a.s.l.
28			Slope	slope	°
29			North aspect	northness of slope aspect	1 if north-exposed, -1 if south-exposed
		snow	Snow duration	median yearly snow cover duration (difference between yearly doy of snowmelt and doy of snow onset)	days

*we derived normalized fluxes by dividing mean daily fluxes in Wm^{-2} by the daily maximum potential incoming shortwave radiation in Wm^{-2}, based on location and topographical conditions[20] and multiplying by 100 (indicated with prefix "n.", unit: %)

Supplementary Table 2

Supplementary Table 2. **Overview of sites used in this study.** Siteid: name of site; network: data distribution network; lat.N: latitude (WGS84) in decimal degrees; lon.E: longitude (WGS84) in decimal degrees; Vegetation type: local vegetation type derived from in situ descriptions (B: barrens; GT: graminoid; P: prostrate-shrub; S: erect-shrub; W: wetlands; GL: glacier; BPB: boreal peat bog; see Methods and ref. column); Vegetation type_d: detailed vegetation type derived from in situ descriptions; CAVM type: landscape-scale dominant vegetation type extracted from CAVM[2] (B: barrens; G: graminoid; P: prostrate-shrub; S: erect-shrub; W: wetlands; GL: glacier; Nar: non-Arctic vegetation); CAVM subzone: bioclimatic subzones extracted from CAVM[2] (A-E, <E and Glaciers); Perm.ext: permafrost extent (C: continuous; D: discontinuous; SI: sporadic or isolated patches; o: ocean/inland seas; g: glaciers); Perm.ice: ground ice content (h: high; m: medium; l: low; o: ocean/inland seas; g: glaciers); Altitude extracted from ref.[21]; Temp.: average mean annual air temperature (1979-2018) extracted from CHELSA V2.1[22,23]; Precip.: average annual precipitation (1979-2018) extracted from CHELSA V2.1[22,24]; Snow.durat.: median yearly snow duration (2000-2020) extracted from MODIS MOD10C1[25]; variables: variables for which data are available; Min year: average start of data time series; Max year: average end of data time series; Correspondence: last name of corresponding data contributors; Ref.: site reference.

Nr	Siteid	Network	Lat.N	Lon.E	Vegetation type	Vegetation type_d	CAVM type	CAVM subzone	Perm.ext	Perm.ice	Altitude (m a.s.l.)	Temp. (°C)	Precip. (mm)	Sow durat. (d)	Variables	Min year	Max year	Correspondence	Ref.
1	KPC_L	PROMICE	79.91	-24.08	GL	GL	GL	Glaciers	g	g	402.33	-17.11	220.21	365	Rnet|H|LE|SWnet|LWnet|Kin|Kout|Lin|Lout|Tair|Tsurf	2008	2019	van As;Fausto;Vandecrux	26,27
2	KPC_U	PROMICE	79.83	-25.17	GL	GL	GL	Glaciers	g	g	902.98	-17.06	235.78	365	Rnet|H|LE|SWnet|LWnet|Kin|Kout|Lin|Lout|Tair|Tsurf	2008	2019	van As;Fausto;Vandecrux	26,27

3	SJ-Blv	FLUXNET	78.92	11.83	P	P1	B1	B	g	g	56.22	-5.27	779.99	288.75	Rnet|H|LE|G|SWnet|LWnet|Kin|Kout|Lin|Lout|Tair|Tsurf	2003	2020	Boike;Westermann;Lüers;Langer;Piel;Bornemann;Grünberg [28,29]
4	SJ-Adv	FLUXNET	78.19	15.92	GT	G1	P2	C	C	m	43.03	-6.36	357.11	276	Rnet|H|LE|G|SWnet|LWnet|Kin|Kout|Lin|Lout|Tair|Tsurf	2012	2014	-Parmentier, Pirk, Mastepanov, Christensen [30]
5	Tunu-N	GC-Net	78.02	-33.99	GL	GL	GL	Glaciers	g	g	2112	-25.27	127.67	365	Rnet|H|LE|G|SWnet|LWnet|Kin|Kout|Lin|Lout|Tair|Tsurf	1998	2017	Vandecrux [31]
6	THU_U	PROMICE	76.42	-68.15	GL	GL	GL	Glaciers	g	g	773.52	-14.81	383.25	365	Rnet|H|LE|SWnet|LWnet|Kin|Kout|Lin|Lout|Tair|Tsurf	2010	2019	van As;Fausto;Vandecrux [26,27]
7	THU_L	PROMICE	76.4	-68.27	GL	GL	GL	Glaciers	g	g	576.59	-14.2	345.81	365	Rnet|H|LE|SWnet|LWnet|Kin|Kout|Lin|L	2010	2019	van As;Fausto [26,27]

															out|Tair|Tsurf			;Vandecrux	
8	EGP	PROMICE	75.62	-35.97	GL	GL	GL	Glaciers	g	g	2705.01	-27.57	132.28	365	Rnet|H|LE|SWnet|LWnet|Kin|Kout|Lin|Lout|Tair|Tsurf	2016	2019	van As;Fausto;Vandecrux	[26,27]
9	NASA-E	GC-Net	75	-30	GL	GL	GL	Glaciers	g	g	2659.14	-25.65	169.94	365	Rnet|H|LE|G|SWnet|LWnet|Kin|Kout|Lin|Lout|Tair|Tsurf	1998	2017	Vandecrux	[31]
10	GL-ZaF	FLUXNET	74.48	-20.55	S	S1	S1	C	C	l	86.89	-10.95	432.15	265	Rnet|H|LE|G|SWnet|Kin|Kout|Tair|Tsurf	2009	2011	Lund;Jackowicz-Korczynski;Abermann	[32]
11	GL-ZaH	FLUXNET	74.47	-20.55	P	P2	S1	C	C	l	82.2	-10.81	423.86	265	Rnet|H|LE|SWnet|Kin|Kout|Tair|Tsurf	2004	2014	Lund;Jackowicz-Korczynski;Abermann	[33]
12	NASA-U	GC-Net	73.84	-49.5	GL	GL	GL	Glaciers	g	g	2367.67	-21	405.3	365	Rnet|H|LE|G|SWnet|LWnet|Kin	1998	2017	Vandecrux	[31]

															|Kout|Lin|Lout|Tair|Tsurf				
13	UPE_L	PROMICE	72.89	-54.3	GL	GL	GL	Glaciers	g	g	238.03	-8.38	490.15	365	Rnet|H|LE|SWnet|LWnet|Kin|Kout|Lin|Lout|Tair|Tsurf	2009	2019	van As;Fausto;Vandecrux	[26,27]
14	UPE_U	PROMICE	72.89	-53.58	GL	GL	GL	Glaciers	g	g	973.07	-11.55	488.56	365	Rnet|H|LE|SWnet|LWnet|Kin|Kout|Lin|Lout|Tair|Tsurf	2009	2019	van As;Fausto;Vandecrux	[26,27]
15	Summit	GC-Net	72.58	-38.5	GL	GL	GL	Glaciers	g	g	3248.92	-27.55	262.89	365	Rnet|H|LE|G|SWnet|LWnet|Kin|Kout|Lin|Lout|Tair|Tsurf	1998	2017	Vandecrux	[31]
16	SCO_U	PROMICE	72.39	-27.23	GL	GL	GL	Glaciers	g	g	1028.46	-15.44	213.34	365	Rnet|H|LE|SWnet|LWnet|Kin|Kout|Lin|Lout|Tair|Tsurf	2008	2019	van As;Fausto;Vandecrux	[26,27]

17	RU-Sam	FLUXNET	72.37	126.5	W	W2	W2	D	o	o	5.58	-13	410.02	252.25	Rnet|H|LE|SWnet|LWnet|Kin|Kout|Lin|Lout|Tair|Tsurf	2002	2014	Kutzbach; Sachs;Boike;Wille;Schreiber;Langer;Runkle;Holl	[34]
18	SCO_L	PROMICE	72.22	-26.82	GL	GL	GL	Glaciers	g	g	515.13	-12.64	243.54	365	Rnet|H|LE|SWnet|LWnet|Kin|Kout|Lin|Lout|Tair|Tsurf	2008	2019	van As;Fausto;Vandecrux	[26,27]
19	RU-Tks	FLUXNET	71.59	128.89	GT	G3	G4	D	o	o	9.9	-12.98	504.19	247.5	Rnet|H|LE|SWnet|Kin|Kout|Tair|Tsurf	2010	2014	Aurela;Laurila;Hatakka;Tuovinen;Rainne	[35]
20	US-A10	Ameriflux	71.32	-156.61	S	S1	G3	C	C	h	2.92	-10.2	242.94	253	Rnet|H|LE|G|SWnet|LWnet|Kin|Kout|Lin|Lout|Tsurf	2011	2019	Sullivan	https://ameriflux.lbl.gov/sites/siteinfo/US-A10

21	US-Brw	Ameriflux	71.32	-156.61	W	W1	G3	C	C	h	3.07	-10.22	277.92	253.5	Rnet|H|LE|G|Tair	1998	2007	Zona;Oechel	[36]
22	US-NGB	Ameriflux	71.28	-156.61	W	W2	G3	C	C	h	2.1	-10.29	275.3	251	Rnet|H|LE|G|SWnet|LWnet|Kin|Kout|Lin|Lout|Tair	2012	2019	Torn	[37]
23	RU-Cok	FLUXNET	70.83	147.49	GT	G4	G4	E	C	h	8.79	-13.16	292.36	246.5	Rnet|H|LE|G|SWnet|LWnet|Kin|Kout|Lin|Lout|Tair|Tsurf	2003	2014	Dolman;van;Parmentier;Belelli;Dean;van;Maximov	[38,39]
24	US-A03	Ameriflux	70.5	-149.88	W	W1	W2	C	C	h	0.48	-10.28	199.63	248.5	Rnet|H|LE|G|SWnet|LWnet|Kin|Kout|Lin|Lout|Tsurf	2014	2019	Sullivan	https://ameriflux.lbl.gov/sites/siteinfo/US-A03
25	US-Atq	Ameriflux--FLUXNET	70.47	-157.41	GT	G4	G4	D	C	h	20.14	-10.19	289.97	256.5	Rnet|H|LE|G|Kin|Tair|Tsurf	2000	2008	Zona;Oechel	[36,40,41]

26	US-Upa	Ameriflux	70.28	-148.88	W	W3	W2	D	C	h	10.06	-10.39	252.84	248	Rnet|H|LE|G|Kin|Tair|Tsurf	1994	1994	Oechel	[42]
27	CP1	GC-Net	69.88	-46.99	GL	GL	GL	Glaciers	g	g	2002.87	-16.79	792.35	365	Rnet|H|LE|G|SWnet|LWnet|Kin|Kout|Lin|Lout|Tair|Tsurf	1998	2017	Vandecrux	[31]
28	US-HVa	Ameriflux	69.14	-148.84	GT	G4	G4	E	C	m	317.04	-10	429.32	238.25	Rnet|H|LE|G|Kin|Tair	1994	1995	Oechel	[42]
29	US-An1	Ameriflux	68.99	-150.28	GT	G4	G4	E	C	m	357.35	-9.47	455.29	242.75	Rnet|H|LE|G|SWnet|LWnet|Kin|Kout|Lin|Lout|Tair	2008	2010	Rocha;Shaver;Hobbie	[43]
30	US-An2	Ameriflux	68.95	-150.21	GT	G4	S1	E	C	m	406.97	-9.43	488.85	239.75	Rnet|H|LE|G|SWnet|LWnet|Kin|Kout|Lin|Lout|Tair	2008	2010	Rocha;Shaver;Hobbie	[43]
31	US-An3	Ameriflux	68.93	-150.27	GT	G4	S1	E	C	m	431.34	-9.42	503.09	247	Rnet|H|LE|G|SWnet|LWnet|Kin	2008	2010	Rocha;Shaver;Hobbie	[43]

<table>
<tr><td></td><td></td><td></td><td></td><td></td><td></td><td></td><td></td><td></td><td></td><td></td><td></td><td></td><td></td><td></td><td>|Kout|Lin|
Lout|Tair</td><td></td><td></td><td></td><td></td></tr>
<tr><td>32</td><td>US-xTL</td><td>Ameriflux</td><td>68.66</td><td>-149.37</td><td>GT</td><td>G4</td><td>G3</td><td>E</td><td>C</td><td>m</td><td>823.23</td><td>-9.74</td><td>542.74</td><td>248.25</td><td>Rnet|H|LE
|G|SWnet|
LWnet|Kin
|Kout|Lin|
Lout|Tair|
Tsurf</td><td>2017</td><td>2019</td><td>-</td><td>https
://a
merif
lux.lb
l.gov
/sites
/sitei
nfo/
US-
xTL-</td></tr>
<tr><td>33</td><td>RU-Che</td><td>FLUXNET</td><td>68.61</td><td>161.34</td><td>W</td><td>W3</td><td>NAr</td><td><E</td><td>C</td><td>h</td><td>8.91</td><td>-10.42</td><td>355.26</td><td>223</td><td>Rnet|H|LE
|G|SWnet|
LWnet|Kin
|Kout|Lin|
Lout|Tair|
Tsurf</td><td>2002</td><td>2005</td><td>Merbold;
Rebmann;
Corradi</td><td>[44,45]</td></tr>
<tr><td>34</td><td>US-ICh</td><td>Ameriflux--
AON</td><td>68.61</td><td>-149.3</td><td>P</td><td>P2</td><td>S1</td><td>E</td><td>C</td><td>l</td><td>946.09</td><td>-9.96</td><td>587.89</td><td>249.5</td><td>Rnet|H|LE
|G|SWnet|
Kin|Kout|T
air</td><td>2007</td><td>2018</td><td>Edgar;Bre
t-
Harte;Eus
kirchen</td><td>[46]</td></tr>
<tr><td>35</td><td>US-ICt</td><td>Ameriflux--
AON</td><td>68.61</td><td>-149.3</td><td>GT</td><td>G4</td><td>S1</td><td>E</td><td>C</td><td>l</td><td>927.88</td><td>-9.96</td><td>587.89</td><td>246.75</td><td>Rnet|H|LE
|G|SWnet|
LWnet|Kin
|Kout|Lin|
Lout|Tair</td><td>2009</td><td>2018</td><td>Edgar;Bre
t-
Harte;Eus
kirchen</td><td>[46]</td></tr>
</table>

36	US-ICs	Ameriflux--AON	68.61	-149.31	W	W2	S1	E	C	I	911.97	-9.66	555.23	246.75	Rnet|H|LE|G|SWnet|Kin|Kout|Tair	2007	2018	Edgar;Bret-Harte;Euskirchen	[46]
37	US-Ivo	Ameriflux--FLUXNET	68.49	-155.75	GT	G4	G4	E	C	I	561.53	-9.95	549.91	250	Rnet|H|LE|G|SWnet|LWnet|Kin|Kout|Lin|Lout|Tair|Tsurf	2003	2012	Zona;Oechel	[36]
38	SE-St1	FLUXNET	68.35	19.05	BPB	BPB	NAr	<E	D	I	383.86	0.54	670.56	222.5	Rnet|H|LE|SWnet|LWnet|Kin|Kout|Lin|Lout|Tair|Tsurf	2012	2014	Friborg;Jammet;Crill	http://www.europe-fluxdata.eu/home/site-details?id=SE-St1
39	FI-Lom	FLUXNET	68	24.21	BPB	BPB	NAr	<E	D	I	300.02	-0.02	769.63	220.5	Rnet|H|LE|G|SWnet|LWnet|Kin|Kout|Lin|Lout|Tair|Tsurf	2007	2009	Aurela;Lohila;Tuovinen;Hatakka;Rainne;Mäkelä;Lauria	[47]

40	KAN_B	PROMICE	67.13	-50.18	B	B3	S1	E	C	l	404.47	-5.31	327.97	#N/A	H|LE|LWnet|Lin|Lout|Tair|Tsurf	2011	2019	van As;Fausto;Vandecrux	[26,27]
41	KAN_L	PROMICE	67.1	-49.95	GL	GL	GL	Glaciers	g	g	691.11	-6.3	364.41	365	Rnet|H|LE|SWnet|LWnet|Kin|Kout|Lin|Lout|Tair|Tsurf	2008	2019	van As;Fausto;Vandecrux	[26,27]
42	KAN_M	PROMICE	67.07	-48.84	GL	GL	GL	Glaciers	g	g	1303.34	-10.48	522.66	365	Rnet|H|LE|SWnet|LWnet|Kin|Kout|Lin|Lout|Tair|Tsurf	2008	2019	van As;Fausto;Vandecrux	[26,27]
43	RU-Vrk	FLUXNET	67.05	62.94	S	S2	S1	E	Sl	m	91.95	-4.93	766.52	231.5	Rnet|H|LE|G|Kin|Tair	2008	2008	Friborg;Biasi;J.	[48]
44	KAN_U	PROMICE	67	-47.03	GL	GL	GL	Glaciers	g	g	1879.35	-14.41	633.97	365	Rnet|H|LE|SWnet|LWnet|Kin|Kout|Lin|Lout|Tair|Tsurf	2009	2019	van As;Fausto;Vandecrux	[26,27]

45	Dye-2	GC-Net	66.48	-46.28	GL	GL	GL	Glaciers	g	g	2160.44	-16.12	626.06	365	Rnet|H|LE|G|SWnet|LWnet|Kin|Kout|Lin|Lout|Tair|Tsurf	1998	2017	Vandecrux	[31]
46	NASA-SE	GC-Net	66.48	-42.5	GL	GL	GL	Glaciers	g	g	2429.69	-18.44	1024.82	365	Rnet|H|LE|G|SWnet|LWnet|Kin|Kout|Lin|Lout|Tair|Tsurf	1998	2017	Vandecrux	[31]
47	Saddle	GC-Net	66	-44.5	GL	GL	GL	Glaciers	g	g	2506.16	-18.58	742.09	365	Rnet|H|LE|G|SWnet|LWnet|Kin|Kout|Lin|Lout|Tair|Tsurf	1998	2017	Vandecrux	[31]
48	TAS_A	PROMICE	65.78	-38.9	GL	GL	GL	Glaciers	g	g	946.58	-6.4	2127.02	365	Rnet|H|LE|SWnet|LWnet|Kin|Kout|Lin|Lout|Tair|Tsurf	2013	2018	van As;Fausto;Vandecrux	[26,27]
49	TAS_U	PROMICE	65.7	-38.87	GL	GL	GL	Glaciers	g	g	615.76	-4.29	1935.28	365	Rnet|H|LE|SWnet|LWnet|Kin|Kout|Lin|L	2008	2015	van As;Fausto;Vandecrux	[26,27]

															out|Tair|Tsurf				
50	MIT	PROMICE	65.69	-37.83	GL	GL	B3	D	g	g	487.31	-3.23	1628.88	365	Rnet|H|LE|SWnet|LWnet|Kin|Kout|Lin|Lout|Tair|Tsurf	2009	2018	van As;Fausto;Vandecrux	[26,27]
51	TAS_L	PROMICE	65.64	-38.9	GL	GL	GL	Glaciers	g	g	307.45	-2.4	1738.13	365	Rnet|H|LE|SWnet|LWnet|Kin|Kout|Lin|Lout|Tair|Tsurf	2007	2019	van As;Fausto;Vandecrux	[26,27]
52	NUK_N	PROMICE	64.95	-49.88	GL	GL	GL	Glaciers	g	g	948.73	-6.73	1101.2	365	Rnet|H|LE|SWnet|LWnet|Kin|Kout|Lin|Lout|Tair|Tsurf	2010	2014	van As;Fausto;Vandecrux	[26,27]
53	NUK_U	PROMICE	64.51	-49.27	GL	GL	GL	Glaciers	g	g	1154.73	-8.37	1075.46	365	Rnet|H|LE|SWnet|LWnet|Kin|Kout|Lin|Lout|Tair|Tsurf	2007	2019	van As;Fausto;Vandecrux	[26,27]

54	NUK_L	PROMICE	64.48	-49.54	GL	GL	B1	E	D	I	564.56	-4.89	925.62	365	Rnet|H|LE|SWnet|LWnet|Kin|Kout|Lin|Lout|Tair|Tsurf	2007	2019	van As;Fausto;Vandecrux	[26,27]
55	NUK_K	PROMICE	64.16	-51.36	GL	GL	B3	D	SI	I	762.4	-3.46	1171.58	263.75	Rnet|H|LE|SWnet|LWnet|Kin|Kout|Lin|Lout|Tair|Tsurf	2014	2019	van As;Fausto;Vandecrux	[26,27]
56	GL-NuF	FLUXNET	64.13	-51.39	W	W3	S1	D	SI	I	73.66	-3.86	1494.9	248.5	Rnet|H|LE|SWnet|Kin|Kout|Tair|Tsurf	2009	2014	-	[49]
57	US-EML	Ameriflux	63.88	-149.25	GT	G4	NAr	<E	D	I	683.46	-3.28	639.69	201.5	Rnet|H|LE|SWnet|LWnet|Kin|Kout|Lin|Lout|Tair|Tsurf	2010	2018	Schuur	[50]
58	US-xHE	Ameriflux	63.88	-149.21	S	S2	NAr	<E	D	I	691.01	-3.39	657.41	206.5	Rnet|H|LE|G|SWnet|LWnet|Kin|Kout|Lin|Lout|Tair|Tsurf	2017	2019	-	https://ameriflux.lbl.gov/sites/siteinfo/

																			US-xHE
59	South Dome	GC-Net	63.15	-44.82	GL	GL	GL	Glaciers	g	g	2930.25	-18.99	1102.71	365	Rnet|H|LE|G|SWnet|LWnet|Kin|Kout|Lin|Lout|Tair|Tsurf	1998	2017	Vandecrux	[31]
60	CA-SCB	Ameriflux	61.31	-121.3	BPB	BPB	NAr	<E	SI	l	269.71	-1.91	613.79	198	Rnet|H|LE|G|SWnet|LWnet|Kin|Kout|Lin|Lout|Tair|Tsurf	2014	2017	Sonnentag;L	[51]
61	QAS_A	PROMICE	61.24	-46.73	GL	GL	GL	Glaciers	g	g	1047.81	-6.12	2216.9	365	Rnet|H|LE|SWnet|LWnet|Kin|Kout|Lin|Lout|Tair|Tsurf	2012	2015	van As;Fausto;Vandecrux	[26,27]
62	QAS_U	PROMICE	61.18	-46.82	GL	GL	GL	Glaciers	g	g	933.52	-5.16	2212.55	365	Rnet|H|LE|SWnet|LWnet|Kin|Kout|Lin|Lout|Tair|Tsurf	2008	2019	van As;Fausto;Vandecrux	[26,27]

63	QAS_M	PROMICE	61.1	-46.83	GL	GL	GL	Glaciers	g	g	670.69	-3.65	2022.87	365	Rnet|H|LE|SWnet|LWnet|Kin|Kout|Lin|Lout|Tair|Tsurf	2016	2019	van As;Fausto;Vandecrux	[26,27]
64	QAS_L	PROMICE	61.03	-46.85	GL	GL	GL	Glaciers	g	g	315.65	-1.88	1657.42	365	Rnet|H|LE|SWnet|LWnet|Kin|Kout|Lin|Lout|Tair|Tsurf	2007	2019	van As;Fausto;Vandecrux	[26,27]

Supplementary Table 3

Supplementary Table 3. **Overview of data sources used in this study.** Network/category: name of data distribution networks for surface energy fluxes/components and environmental categories for SEB-drivers, Variables: variables used in this study that are based on corresponding data sources, Time of retrieval: time when data was retrieved by authors, Times covered: timespan covered by data, Resolution: spatial and temporal resolution, respectively (if applicable), Product name/source: name, version and link to data product, Reference: data product reference. See Methods and Supplementary Table 1 for a more detailed description of surface energy fluxes/components and drivers.

Nr	Network / category	Variables	Time of retrieval	Times covered	Resolution	Product name/source	Reference
1	AmeriFlux	surface energy fluxes and components	13.05.2020	1994-2019	half-hourly / hourly	product: AMF_(.+)_BASE_HH(HR) / https://ameriflux.lbl.gov/login/?redirect_to=/data/download-data/	[52]
2	AON	surface energy fluxes and components	04.08.2020	2007-2019	half-hourly	http://aon.iab.uaf.edu/data_access, contributed by: Edgar, C.	[53,54]
3	FLUXNET (includes GEM and ICOS)	surface energy fluxes and components	13.05.2020	1999-2019	half-hourly / hourly	product: FLUXNET2015_FULLSET_HH(HR) / https://fluxnet.org/data/download-data/	[55]
4	GC-Net	surface energy fluxes and components	11.05.2020	1998-2017	hourly	product: hourly / http://cires1.colorado.edu/steffen/gcnet/order/admin/station.php, contributed by: Vandecrux, B.	[31,56]
5	PROMICE	surface energy fluxes and components	29.05.2020	2007-2019	hourly	Product: hourly version 3 (v3) / at https://promice.org/PromiceDataPortal/api/download/f24019f7-d586-4465-8181-d4965421e6eb/v03/hourly/csv	[26,27]
6	vegetation	CAVM type	19.09.2019	1993-2018	1 km	Raster CAVM: raster_cavm_v1 / https://data.mendeley.com/datasets/c4xj5rv6kv/1	[2]
7	climate	CAVM subzone	06.08.2021	1993-2003	1:7,500,000	Product: cp_biozone_la_shp / http://www.arcticatlas.org/maps/themes/cp/cpbz	[57]
		Temperature, Precipitation, Snow amount, Continentality, Summer warmth	30.09.2020	1979-2018	30 arc seconds (~500m at 60°N), daily	CHELSA V2.1 submodel PMIP3 products: mean daily air temperature [K/10]: “tas”, daily precipitation amount [kg m^{-2} day^{-1}/10]: “pr”/ https://chelsa-climate.org/, contributed by: Karger, D. N.	[22–24]
8	snow	Snow duration	24.07.2020	2000-2020	0.05° (~ 3 km at 60°N), daily	MODIS/Terra Snow Cover Daily L3 Global 0.05Deg CMG, Version 6 product: “MOD10C1” / https://nsidc.org/data/MOD10C1/versions/6#0, contributed by: Grünberg, I.	[25]
9	clouds	Cloud cover<br>Cloud temperature	10.09.2020	1984-2016	1°,(~56 km at 60°N), monthly	ISCCP-Basic-H series: ISCCP-Basic.HGM.v01r00.GLOBAL products: “cldamt”,”tc” / https://www.ncei.noaa.gov/data/international-satellite-cloud-climate-project-isccp-h-series-data/access/isccp-basic/	[58,59]
10	permafrost	Permafrost extent<br>Permafrost ice content	09.09.2020	1997	1:10,000,000	NSIDC gdd318_map_circumArctic version 2: product: “permaice.shp” / ftp://sidads.colorado.edu/pub/DATASETS/fgdc/ggd318_map_circumArctic/	[60]
11	topography	Altitude, Slope, North aspect	02.09.2020	2016-2018	100 m	ArcticDEM: Arcticdem_mosaic_100m_v3.0 / http://data.pgc.umn.edu/elev/dem/setsm/ArcticDEM/mosaic/v3.0/100m/	[21]

Supplementary Table 4

Supplementary Table 4. **Significant post-hoc pairwise comparisons of summer surface energy flux magnitudes among glacier surfaces and vegetation types.** In order to assess the magnitude and significance of differences in summer surface energy fluxes among vegetation types and glacier surfaces (Vegetation type variable), we conducted a post-hoc pairwise comparison analysis for all linear mixed-models in Table 1 (bonferroni-corrected P-values; nr. of sites: 64, nr. of site years: 652, see Methods). R_{net}: net radiation, SW_{net}: net shortwave radiation, LW_{net}: net longwave radiation, H: sensible heat flux, LE: latent heat flux, G: ground heat flux. Vegetation types: B: barrens; BPB: boreal peat bog; GT: graminoid; P: prostrate-shrub; S: erect-shrub; W: wetlands; GL: glacier. Significant differences among vegetation types (i.e. not involving comparison to glacier) are highlighted with gray background color. Note that flux direction convention is positive away from the surface for non-radiative surface energy fluxes (H, LE and G).

Season	Surface energy flux	Pairwise comparison	Estimated difference (Wm^{-2})	Estimated standard error (Wm^{-2})	Z-value	P-value	P-star
JJA	Rnet	GL - BPB	-84.5	25.1	-3.4	0.012	*
JJA	H	P - GL	62.9	16.4	3.8	0.003	**
JJA	H	S - GL	62.1	16.5	3.8	0.003	**
JJA	H	P - B	57.6	18.6	3.1	0.041	*
JJA	H	W - GL	57.4	15.9	3.6	0.007	**
JJA	H	S - B	56.9	18.7	3.0	0.049	*
JJA	H	GL - GT	-49.5	15.9	-3.1	0.040	*
JJA	LE	BPB - B	74.9	13.4	5.6	0.000	***
JJA	LE	GL - BPB	-66.8	11.3	-5.9	0.000	***
JJA	LE	P - BPB	-51.0	10.1	-5.1	0.000	***
JJA	LE	S - BPB	-50.5	12.0	-4.2	0.001	***
JJA	LE	GT - B	46.6	11.6	4.0	0.001	**
JJA	LE	W - BPB	-43.0	9.2	-4.7	0.000	***
JJA	LE	GL - GT	-38.5	9.1	-4.2	0.000	***
JJA	LE	GT - BPB	-28.3	8.7	-3.3	0.023	*
JJA	G	S - GL	10.7	3.2	3.3	0.014	*
JJA	SWnet	GL - BPB	-103.5	25.2	-4.1	0.001	***
JJA	SWnet	W - GL	69.2	23.4	3.0	0.047	*
JJA	SWnet	GL - GT	-67.3	22.2	-3.0	0.036	*
JJA	LWnet	S - B	42.3	10.5	4.0	0.001	**
JJA	LWnet	GL - B	34.7	8.2	4.2	0.000	***
JJA	LWnet	W - B	32.0	9.6	3.3	0.017	*
JJA	LWnet	S - GT	20.2	6.4	3.1	0.034	*

Supplementary Table 5

Supplementary Table 5. **Vegetation type effect significance and estimated mean ± 95% confidence intervals (CI) for normalized surface energy fluxes and the vegetation subset of data excluding glacier sites.** Vegetation type effect significance is derived from a mixed-model analysis with the vegetation subset of data (nr. of sites: 31, nr. of site years: 234) and monthly averaged surface energy flux magnitudes, summarized by ANOVA (F-value, P-value: ***:P<0.001, **: P<0.01, *:P<0.05, n.s.: not significant). Mean estimates ± 95% CI are given for the Vegetation types boreal peat bog, graminoid, prostrate-shrub, and erect-shrub tundra; wetland and barren complexes across June, July and August. Estimates for surface energy fluxes are normalized, i.e. expressed as percentage (%) of maximum potential incoming shortwave radiation (Methods), as indicated by the "n."-prefix. for R_{net}: net radiation; H: sensible heat flux; LE: latent heat flux; G: ground heat flux; SW_{net}: net shortwave radiation; LW_{net}: net longwave radiation. Estimates for albedo and T_{surf} (surface temperature) are unitless and in °C, respectively. Note: flux direction convention is positive away from the surface for heat fluxes (i.e. H, LE and G). Vegetation type effect significance P<0.05 is highlighted with gray shading.

**sea-son**	**surface energy flux**	**unit**	**F-value**	**Boreal peat bog**	**Wetland complex**	**Graminoid tundra**	**Erect-shrub tundra**	**Prostrate-shrub tundra**	**Barren complex**
June	n.Rnet	%	$F_{4,23}$=2.2 n.s.	18.8±4.6	17.5±4.5	16.2±4.4	14.9±5.5	13.6±5.8	-
	n.SWnet	%	$F_{4,18}$=1.5 n.s.	25.7±4.6	23.8±4.6	23.7±4.3	20.0±6.1	22.5±5.7	-
	n.LWnet	%	$F_{5,12}$=3.6*	-6.6±2.7	-5.8±2.5	-6.8±2.3	-4.5±2.7	-5.1±3.1	-9.5±2.2
	n.H	%	$F_{5,17}$=3.4*	0.9±5.6	5.4±4.7	4.6±4.7	5.0±4.9	3.9±4.9	-2.8±4.3
	n.LE	%	$F_{5,12}$=4.0*	11.9±3.8	4.9±3.0	5.6±2.9	4.8±3.1	3.1±3.1	0.3±2.7
	n.G	%	$F_{4,15}$=0.9 n.s.	0.9±0.9	1.8±1.1	1.9±1.0	2.3±1.2	1.6±1.3	-
	albedo	-	$F_{4,18}$=3.6*	0.13±0.14	0.24±0.14	0.19±0.13	0.29±0.18	0.33±0.17	-
	Tsurf	°C	$F_{5,14}$=2.8.	11.9±9.2	3.6±8.9	5.1±8.7	5.2±9.2	3.6±9.5	-4.0±7.6
July	n.Rnet	%	$F_{4,22}$=2.9*	17.4±5.5	15.6±4.7	13.2±4.6	14.9±5.7	15.2±6.2	-
	n.SWnet	%	$F_{4,18}$=1.7 n.s.	24.1±5.3	22.0±4.7	20.1±4.5	20.4±6.4	24.1±6.0	-
	n.LWnet	%	$F_{5,13}$=2.3 n.s.	-6.5±2.4	-5.6±2.3	-6.2±2.2	-4.7±2.5	-6.3±2.9	-9.5±2.1
	n.H	%	$F_{5,16}$=4.4*	2.7±5.4	5.8±4.2	4.7±4.1	7.2±4.5	6.3±4.4	-2.3±3.8
	n.LE	%	$F_{5,10}$=12.2***	9.8±2.4	5.6±1.9	6.6±1.9	5.7±2.4	5.1±2.0	-0.2±1.7
	n.G	%	$F_{4,16}$=1.7 n.s.	0.6±1.0	1.7±1.1	1.6±1.1	2.5±1.2	1.9±1.4	-
	albedo	-	$F_{4,18}$=1.6 n.s.	0.14±0.16	0.18±0.14	0.18±0.13	0.20±0.19	0.16±0.18	-
	Tsurf	°C	$F_{5,13}$=3.7*	14.2±7.8	7.9±7.5	9.0±7.4	11.1±8.2	9.4±8.1	-3.3±6.5
August	n.Rnet	%	$F_{4,20}$=0.4 n.s.	10.5±5.5	9.2±5.2	9.6±5.1	8.1±6.0	8.8±6.2	-
	n.SWnet	%	$F_{4,14}$=0.5 n.s.	17.4±5.8	16.2±5.4	15.4±5.1	12.8±6.9	17.1±6.1	-
	n.LWnet	%	$F_{5,9}$=1.0 n.s.	-6.3±3.3	-4.9±3.2	-5.2±3.1	-4.4±3.4	-6.8±4.2	-7.5±2.7
	n.H	%	$F_{5,13}$=4.3*	1.0±3.3	3.2±2.6	3.1±2.5	3.7±2.7	3.3±2.7	-1.9±2.4
	n.LE	%	$F_{5,7}$=12.4**	8.7±2.5	3.9±1.9	4.6±1.9	2.2±2.5	3.2±2.0	0.0±1.7
	n.G	%	$F_{4,15}$=3.5*	0.1±0.6	0.9±0.6	1.1±0.6	1.6±0.8	0.8±0.8	-
	albedo	-	$F_{4,14}$=2.4 n.s.	0.12±0.21	0.17±0.20	0.19±0.19	0.21±0.26	0.16±0.23	-
	Tsurf	°C	$F_{5,13}$=4.9**	12.7±9.4	7.2±9.1	6.8±8.9	8.4±9.9	6.4±9.7	-4.4±7.8

Supplementary Table 6

Supplementary Table 6. **Confusion matrix: detailed local-scale vegetation type based on in situ descriptions (detailed Vegetation type variable; rows) and corresponding landscape-scale dominant CAVM type (columns).** In situ vegetation types at the local scale were derived for each study site from references listed in Supplementary Table 2 according to a fixed protocol (Methods). Dominant CAVM types in circular areas with radius 500m around the study sites were extracted from the CAVM raster map[2]. CAVM types integrate environmental conditions and dominant vegetation at larger spatial scales that do not always correspond to in situ vegetation types. B1: cryptogam, herb barren; B3: non-carbonate mountain complex; G1: graminoid, forb, cryptogam tundra; G3: non-tussock sedge, dwarf-shrub, moss tundra; G4: tussock-sedge, dwarf-shrub, moss tundra; P1: prostrate dwarf-shrub, herb, lichen tundra; P2: prostrate/hemi-prostrate dwarf-shrub, lichen tundra; S1: erect dwarf-shrub, moss tundra; S2: low-shrub, moss tundra; W1: sedge/grass, moss wetland complex; W2: sedge, moss, dwarf-shrub wetland complex; W3: sedge, moss, low-shrub wetland complex; BPB: boreal peat bog; Nar: non-Arctic vegetation; GL: glacier.

	CAVM type								
Veg. type detail	B1	B3	G3	G4	GL	NAr	P2	S1	W2
B3	0	0	0	0	0	0	0	1	0
BPB	0	0	0	0	0	3	0	0	0
G1	0	0	0	0	0	0	1	0	0
G3	0	0	0	1	0	0	0	0	0
G4	0	0	1	5	0	1	0	3	0
GL	1	2	0	0	30	0	0	0	0
P1	1	0	0	0	0	0	0	0	0
P2	0	0	0	0	0	0	0	2	0
S1	0	0	1	0	0	0	0	1	0
S2	0	0	0	0	0	1	0	1	0
W1	0	0	1	0	0	0	0	0	1
W2	0	0	1	0	0	0	0	1	1
W3	0	0	0	0	0	1	0	1	1

Supplementary Table 7

Supplementary Table 7. **Confusion matrix: vegetation type based on in situ descriptions (Vegetation type variable; rows) and corresponding bioclimatic subzone (CAVM subzone) classes (columns).** In situ vegetation types: B: barrens; BPB: boreal peat bog; GT: graminoid; P: prostrate-shrub; S: erect-shrub; W: wetlands; GL: glacier. Bioclimatic subzone classes (derived from ref.[57]) are based on Arctic phytogeographic zones[61], dominant growth forms and summer temperatures[62,63]. Subzones A-E are therefore generally well aligned with summer warmth index (SWI) classes: Subzone A: 0.1–5 °C, Subzone B: 5–14 °C, Subzone C: 14–22 °C, Subzone D: 22–32 °C, Subzone E: 32–45 °C (values taken from ref.[63]). Subzone A (coldest subzone) was not present in our dataset.

	CAVM subzone					
Veg. type	<E	E	D	C	B	Glaciers
B	0	1	0	0	0	0
BPB	3	0	0	0	0	0
GT	1	8	2	1	0	0
GL	0	1	2	0	0	30
P	0	1	0	1	1	0
S	1	1	0	2	0	0
W	1	1	3	3	0	0

Supplementary Table 8

Supplementary Table 8. **Confusion matrix: vegetation types based on in situ descriptions (Vegetation type variable; rows) and corresponding permafrost extent (columns).** In situ vegetation types: B: barrens; BPB: boreal peat bog; GT: graminoid; P: prostrate-shrub; S: erect-shrub; W: wetlands; GL: glacier. Permafrost extent (derived from ref.[60]) consists of the following classes: C: continuous permafrost (90-100% extent); D: discontinuous permafrost (50- 90% extent); SI: sporadic or isolated patches of permafrost (50% extent); o: ocean/inland seas, and g: glaciers.

	Permafrost extent				
Veg. type	o	SI	D	C	g
B	0	0	0	1	0
BPB	0	1	2	0	0
GT	1	0	1	10	0
GL	0	1	1	0	31
P	0	0	0	2	1
S	0	1	1	2	0
W	1	1	0	6	0

Supplementary Table 9

Supplementary Table 9. **Confusion matrix: vegetation types based on in situ descriptions (Vegetation type variable; rows) and corresponding Permafrost ice content (columns).** In situ vegetation types: B: barrens; BPB: boreal peat bog; GT: graminoid; P: prostrate-shrub; S: erect-shrub; W: wetlands; GL: glacier. Permafrost ground ice content (derived from ref.[60]) consists of the following classes: h: high (>20% ice content); m: medium (10-20% ice content); l: low (0-10% ice content); o: ocean/inland seas, and g: glaciers.

	Permafrost ice content				
Veg. type	o	l	m	h	g
B	0	1	0	0	0
BPB	0	3	0	0	0
GT	1	3	6	2	0
GL	0	2	0	0	31
P	0	2	0	0	1
S	0	2	1	1	0
W	1	2	0	5	0

Supplementary Discussion

Literature synthesis

To assess the status of current knowledge on the Arctic land surface energy budget (SEB), we conducted a systematic literature review according to the PRISMA (Preferred Reporting Items for Systematic reviews and Meta-Analyses) statement and formulated eligibility criteria according to the PICOS (participants, interventions, comparators, outcomes, and study design) approach [ref.[1]; prisma-statement.org; cf. Supplementary Figure 7]. Specifically, we conducted a search on all ISI Web of Science databases on 30 July 2019. The full keyword search string was as follows: TS=(surface AND (((energy OR radiation OR radiative) NEAR/2 (flux* OR budget$ OR *balance$ OR exchange$))) AND (Arctic OR tundra OR "high-latitude")); TI=(NOT (ocean OR "sea-ice" OR glacier)); Language= all languages; Document type= article. The search included all available years and yielded 993 publications published between 1961-2019. From these 993 publications, we excluded all that (i) did not cover terrestrial land areas (e.g. studies of marine and ocean surfaces, or studies of extra-terrestrial energy fluxes), (ii) were conceptual studies without measured data, (iii) did not report on any of the surface energy fluxes we deemed as essential (listed in Supplementary Table 1), (iv) were not peer-reviewed research articles, (v) were not primary research articles (e.g. meta-analyses and reviews), (vi) that only considered times before 1950. The resulting set of 197 studies covering 486 locations was re-examined, and articles were excluded if (vii) they only covered locations below 60° N and (viii) did not cover essential surface energy flux measurements over vegetation or glacier sites. This yielded a final set of 148 studies reporting on 358 locations between 1974-2019 (Supplementary Figures 1, 7).

Among others, we assessed the covered locations, years, standard meteorological seasons, surface energy flux variables, SEB-drivers (Supplementary Figure 8) and the description of the local in situ vegetation type for each study. We classified these in situ vegetation types ('Vegetation type' variable) according to the most similar vegetation classes described in the circumpolar arctic vegetation map (CAVM[2]) according to the decision chain described in the "Vegetation type"-section of the Methods section of the main manuscript.

The majority of studies (82%) simultaneously assessed several surface energy fluxes/components and drivers (Supplementary Table 1; average number of assessed surface energy flux/component variables and drivers per study are 5 and 8, respectively, Supplementary Figure 8). Unfortunately, most studies present their results in a form that is not suitable for a quantitative literature synthesis such as meta-analysis. Specifically, studies either only qualitatively describe differences among in situ SEB-measurements at different times and places (without reporting averages, effect sizes or similar statistical metrics) or use in situ SEB-measurements for model validation only (reporting on RMSE and correlation

coefficients with modeled data but without the actual magnitudes of surface energy fluxes or correlations of drivers and responses). Hence, these studies provide results where the actual magnitudes and seasonalities of surface energy fluxes in dependence of SEB-drivers are difficult to assess without asking study authors for the underlying source data. Therefore, we used the collected literature data only to assess the coverage in space, time and vegetation types across the circumpolar land north of 60° latitude (Supplementary Figure 1) as well as to identify important SEB-drivers (Supplementary Figure 8, 9). We suggest that future studies could include statistical metrics of effects of investigated drivers on responses (e.g. estimated coefficients, correlation coefficients, t-test statistics, F-ratios, etc.; e.g. ref.[3]), in order to facilitate potential reuse and synthesis efforts.

Representativeness of literature and SEB observations

To qualitatively evaluate the representativeness of our results and gaps in current knowledge on Arctic surface energy budgets, we assessed and compared the coverage of study locations in the collected literature and SEB-data (see Methods, Data processing section) in space, time and Vegetation type. Specifically, for all study sites in the collected literature data (148 studies reporting on 358 locations; see above) and SEB-data (nr. sites=64, nr. site years=652), we extracted the geographic locations (WGS84 latitude and longitude in decimal degrees), the years covered by studies and in situ observations of surface energy flux variables (Supplementary Figure 1a), as well as the number of study sites per Vegetation type (Supplementary Figure 1b,c).

We find similar spatial coverage of study sites in the SEB-data and in the collected literature data. Both lack coverage in the western parts of Siberia (especially in the north of Yamal-Nenets and the Krasnoyarsk territories), in the eastern Siberian region of Chukotka and large parts of the Canadian Arctic Archipelago, specifically in the Province of Nunavut and the northern part of Québec (Supplementary Figure 1a). Even though our SEB-data did not cover all in situ observations of surface energy fluxes that currently exist[4], these patterns of spatial coverage align with a more exhaustive evaluation of the representativeness of pan-Arctic eddy-covariance site network[4], as well as previous assessments on the state of sampling in published studies across environmental disciplines in the Arctic[5,6].

Furthermore, we find that most Arctic vegetation types (CAVM[2]) are only represented by a few sites in the SEB-data: all vegetation types except the „G4“ type (tussock-sedge, dwarf-shrub, moss tundra) are represented within 3 or less sites (Supplementary Figure 1b). Several Arctic vegetation types are not contained in the SEB-data; namely “B1“, “B2a“, “B2b“, “B4“ (barren complexes) and „G2“ (graminoid, prostrate dwarf-shrub, forb, moss tundra)[2]. Barren complexes are especially dominant in the Canadian Arctic, cover around one fifth of the terrestrial Arctic area and are characterized by the dominance of non-vascular plant species,

such as lichens and bryophytes[2]. These cryptogams have unique biophysical and hydrological properties exerting effects on surface energy fluxes distinct from other vegetation types[7–9]. For example, lichens have a relatively high shortwave reflectance, affecting albedo and therefore the total absorbed radiative energy at the surface[9]. Additionally, mosses and lichens have a relatively low thermal conductivity, which decreases ground heat fluxes compared to other vegetation types[8,9]. Furthermore, mosses and lichens lack a stomatal regulation of water vapor fluxes, and it has been shown that moss presence can increase latent heat flux depending on soil moisture conditions[8]. Finally, moss and lichen vegetation height is comparatively low, which affects the thickness and thus thermal insulation of snow cover, with consequences for soil moisture and ground heat fluxes in winter and the following spring period[10,11]. Hence, we find barren complex, and more generally, cryptogam-dominated vegetation communities to be underrepresented in the current SEB literature and in situ observations and suggest that they deserve more attention in future studies[7,12].

Finally, the median timespan covered by study site measurements is, unsurprisingly, higher for the SEB-data (11 years) than the literature data (3 years; Supplementary Figure 1a). In the case of the SEB-data, vegetation sites often lack observations in autumn and winter seasons, which hampers robust annual estimates of the SEB and SEB closure, a common issue of interest in many studies of the SEB[13,14]. The median timespan covered by site-measurements is approximately doubled in case of glacier sites (12 years) compared to vegetation sites (6 years). Nevertheless, these time spans are both well below the timespan typically required to quantify trends and climatology of atmospheric state variables (i.e. ~30 years). Therefore, we emphasize the importance of the continued maintenance of existing in situ SEB observations for supporting climate modeling, at best throughout the year, even though we acknowledge the difficulties involved[15–17].

Supplementary References

1. Liberati, A. *et al.* The PRISMA statement for reporting systematic reviews and meta-analyses of studies that evaluate health care interventions: explanation and elaboration. *J. Clin. Epidemiol.* **62**, e1-34 (2009).
2. Raynolds, M. K. *et al.* A raster version of the Circumpolar Arctic Vegetation Map (CAVM). *Remote Sens. Environ.***232**, 111297 (2019).
3. Rosenthal, R. Parametric measures of effect size. In: *The Handbook of research synthesis* (eds. Cooper, H. & Hedges, L. V.) 231–244 (Russell Sage Foundation, 1994).
4. Pallandt, M. *et al.* Representativeness assessment of the pan-Arctic eddy-covariance site network, and optimized future enhancements. *Biogeosci. Discuss.*1-42 (2021). doi:10.5194/bg-2021-133.
5. Virkkala, A. M., Abdi, A. M., Luoto, M. & Metcalfe, D. B. Identifying multidisciplinary research gaps across Arctic terrestrial gradients. *Environ. Res. Lett.* **14**, 124061 (2019).
6. Metcalfe, D. B. *et al.* Patchy field sampling biases understanding of climate change impacts across the Arctic. *Nat. Ecol. Evol.* **2**, 1443–1448 (2018).
7. Sulman, B. N. *et al.* Integrating Arctic Plant Functional Types in a Land Surface Model Using Above- and Belowground Field Observations. *J. Adv. Model. Earth Syst*. **13**, (2021).
8. Blok, D. *et al.* The cooling capacity of mosses: controls on water and energy fluxes in a siberian tundra site. *Ecosystems* **14**, 1055–1065 (2011).
9. Beringer, J., Lynch, A. H., Chapin, F. S., Mack, M. & Bonan, G. B. The representation of arctic soils in the land surface model: the importance of mosses. *J. Clim.* **14**, 3324–3335 (2001).
10. Loranty, M. M., Goetz, S. J. & Beck, P. S. A. Tundra vegetation effects on pan-Arctic albedo. *Environ. Res. Lett.* **6**(2), 024014 (2011).
11. Stoy, P. C., Street, L. E., Johnson, A. V., Prieto-Blanco, A., & Ewing, S. A. Temperature, heat flux, and reflectance of common subarctic mosses and lichens under field

conditions: might changes to community composition impact climate-relevant surface fluxes? *Arct., Antarc., Alp. Res.* **44**(4), 500-508 (2012).

12. Wullschleger, S. D. *et al.* Plant functional types in Earth system models: past experiences and future directions for application of dynamic vegetation models in high-latitude ecosystems. *Ann. Bot.* **114**, 1–16 (2014).
13. Mauder, M., Foken, T. & Cuxart, J. Surface-Energy-Balance Closure over Land: A Review. *Boundary Layer Meteorol.* **177**, 395–426 (2020).
14. Foken, T. The energy balance closure problem: an overview. *Ecol. Appl.* **18**, 1351–1367 (2008).
15. Duncan, B. N. *et al.* Space-based observations for understanding changes in the arctic-boreal zone. *Rev. Geophys.* **58**, (2020).
16. Bartsch, A., Höfler, A., Kroisleitner, C. & Trofaier, A. Land Cover Mapping in Northern High Latitude Permafrost Regions with Satellite Data: Achievements and Remaining Challenges. *Remote Sens.* **8**(12), 979 (2016).
17. Baldocchi, D. Measuring fluxes of trace gases and energy between ecosystems and the atmosphere - the state and future of the eddy covariance method. *Glob. Chang. Biol.* **20**, 3600–3609 (2014).
18. Sellers, P. J. *et al.* Modeling the exchanges of energy, water, and carbon between continents and the atmosphere. *Science* **275**, 502–509 (1997).
19. Bonan, G. Surface Energy Fluxes. In*: Ecological Climatology: Concepts and Applications.* 3rd edn, 193-208 (Cambridge University Press, 2015).
20. Seyednasrollah, B. Solrad: To calculate solar radiation and related variables based on location, time and topographical conditions (v0.99.0). Zenodo (2018) doi:10.5281/zenodo.1249673.
21. Porter, C. *et al.* “ArcticDEM“. V3 [02.09.2020] *Harvard Dataverse* (2018). doi:10.7910/dvn/ohhukh.
22. Karger D. N. *et al.* Data from: Climatologies at high resolution for the earth’s land surface areas. (2017).

23. Karger, D. N. *et al.* Climatologies at high resolution for the earth's land surface areas. *Sci. Data* **4**, 170122 (2017). doi: 10.1038/sdata.2017.122

24. Karger, D. N., Wilson, A. M., Mahony, C., Zimmermann, N. E., & Jetz, W. Global daily 1 km land surface precipitation based on cloud cover-informed downscaling. *Sci. Data* **8**(1), 1-18 (2021).

25. Hall, D. K., Riggs G., A., Solomonson, V. & Sips, N. M. MODIS/Terra Snow Cover Daily L3 Global 0.05Deg CMG. *NASA National Snow and Ice Data Center DAAC* (2015) doi:10.5067/modis/mod10c1.006.

26. Fausto, R. S. *et al.* Programme for Monitoring of the Greenland Ice Sheet (PROMICE) automatic weather station data. *Earth Syst. Sci. Data* **13**, 3819–3845 (2021).

27. Fausto, R. S., van As, D. & Mankoff, D. Programme for monitoring of the Greenland ice sheet (PROMICE): Automatic weather station data. Version: v03, Dataset published via Geological Survey of Denmark and Greenland. (2019).

28. Boike, J., Roth, K. & Ippisch, O. Seasonal snow cover on frozen ground: Energy balance calculations of a permafrost site near Ny-Alesund, Spitsbergen. *J. Geophys. Res. Atmos.* **108**, (2003).

29. Boike, J. *et al.* A 20-year record (1998–2017) of permafrost, active layer and meteorological conditions at a high Arctic permafrost research site (Bayelva, Spitsbergen). *Earth Syst. Sci. Data* **10**, 355–390 (2018).

30. Pirk, N. *et al.* Spatial variability of CO_2 uptake in polygonal tundra: assessing low-frequency disturbances in eddy covariance flux estimates. *Biogeosciences* **14**, 3157–3169 (2017).

31. Vandecrux, B. *et al.* Firn cold content evolution at nine sites on the Greenland ice sheet between 1998 and 2017. *J. Glaciol.* **66**, 591–602 (2020).

32. Stiegler, C., Lund, M., Christensen, T. R., Mastepanov, M. & Lindroth, A. Two years with extreme and little snowfall: effects on energy partitioning and surface energy exchange in a high-Arctic tundra ecosystem. *The Cryosphere* **10**, 1395–1413 (2016).

33. Lund, M., Hansen, B. U., Pedersen, S. H., Stiegler, C. & Tamstorf, M. P. Characteristics

of summer-time energy exchange in a high Arctic tundra heath 2000–2010. *Tellus B: Chem. Phys. Meteorol.* **66**(1), 21631 (2014).

34. Boike, J., Wille, C. & Abnizova, A. Climatology and summer energy and water balance of polygonal tundra in the Lena River Delta, Siberia. *J. Geophys. Res.: Biogeosci.* **113**(G3) (2008).

35. Kodama, Y. *et al.* Wind direction dependency of water and energy fluxes and synoptic conditions over a tundra near Tiksi, Siberia. *Hydrol. Process.* **21**, 2028–2037 (2007).

36. Davidson, S. J. *et al.* Vegetation type dominates the spatial variability in CH4 emissions across multiple arctic tundra landscapes. *Ecosystems* **19**, 1116–1132 (2016).

37. Raz-Yaseef, N. *et al.* Evapotranspiration across plant types and geomorphological units in polygonal Arctic tundra. *J. Hydrol.* **553**, 816–825 (2017).

38. van der Molen, M. K. *et al.* The growing season greenhouse gas balance of a continental tundra site in the Indigirka lowlands, NE Siberia. *Biogeosci.* **4**(6), 985-1003 (2007). doi:10.5194/bg-4-985-2007

39. Dean, J. F. *et al.* East Siberian Arctic inland waters emit mostly contemporary carbon. *Nat. Commun.* **11**, 1627 (2020).

40. Oechel, W. C., Laskowski, C. A., Burba, G., Gioli, B., & Kalhori, A. A. Annual patterns and budget of CO_2 flux in an Arctic tussock tundra ecosystem. J. Geophys. Res.: *Biogeosci.* **119**(3), 323-339 (2014).

41. Kwon, H.-J., Oechel, W. C., Zulueta, R. C. & Hastings, S. J. Effects of climate variability on carbon sequestration among adjacent wet sedge tundra and moist tussock tundra ecosystems. *J. Geophys. Res.: Biogeosci.* **111**(G3) (2006).

42. Oechel, W. C., Vourlitis, G. L., Brooks, S., Crawford, T. L. & Dumas, E. Intercomparison among chamber, tower, and aircraft net CO2 and energy fluxes measured during the Arctic System Science Land-Atmosphere-Ice Interactions (ARCSS-LAII) Flux Study. *J. Geophys. Res. Atmos.* **103**, 28993–29003 (1998).

43. Rocha, A. V. & Shaver, G. R. Burn severity influences postfire CO_2 exchange in arctic tundra. *Ecol. Appl.* **21**, 477–489 (2011).

44. Göckede, M. *et al.* Shifted energy fluxes, increased Bowen ratios, and reduced thaw depths linked with drainage-induced changes in permafrost ecosystem structure. *The Cryosphere* **11**, 2975–2996 (2017).

45. Kittler, F. *et al.* Long-Term Drainage Reduces CO_2 Uptake and CH_4 Emissions in a Siberian Permafrost Ecosystem. *Glob. Biogeochem. Cycles* **31**(12), 1704-1717 (2017).

46. Euskirchen, E. S., Bret-Harte, M. S., Scott, G. J., Edgar, C. & Shaver, G. R. Seasonal patterns of carbon dioxide and water fluxes in three representative tundra ecosystems in northern Alaska. *Ecosphere* **3**(1), 1-19 (2012).

47. Aurela, M. *et al.* Carbon dioxide and energy flux measurements in four northern-boreal ecosystems at Pallas. *Boreal Environ. Res.* **20**, 455–473 (2015).

48. Marushchak, M. E. *et al.* Methane dynamics in the subarctic tundra: combining stable isotope analyses, plot- and ecosystem-scale flux measurements. *Biogeosci.* **13**, 597–608 (2016).

49. Westergaard-Nielsen, A., Lund, M., Hansen, B. U. & Tamstorf, M. P. Camera derived vegetation greenness index as proxy for gross primary production in a low Arctic wetland area. *ISPRS J. Photogramm. Remote Sens.* **86** 89-99 (2013). doi:10.1016/j.isprsjprs.2013.09.006.

50. Belshe, E. F., Schuur, E. A. G., Bolker, B. M. & Bracho, R. Incorporating spatial heterogeneity created by permafrost thaw into a landscape carbon estimate. *J. Geophys. Res.: Biogeosci.* **117**, (2012).

51. Helbig, M. *et al.* The positive net radiative greenhouse gas forcing of increasing methane emissions from a thawing boreal forest-wetland landscape. *Glob. Chang. Biol.* **23**, 2413–2427 (2017).

52. Novick, K. A. *et al.* The AmeriFlux network: A coalition of the willing. *Agric. For. Meteorol.* **249**, 444–456 (2018).

53. Euskirchen, E. S. *et al.* Interannual and Seasonal Patterns of Carbon Dioxide, Water, and Energy Fluxes From Ecotonal and Thermokarst-Impacted Ecosystems on Carbon-Rich Permafrost Soils in Northeastern Siberia. *J. Geophys. Res.: Biogeosci.* **122**, 2651–2668

(2017).

54. Euskirchen, E. S., Bret-Harte, M. S., Shaver, G. R., Edgar, C. W. & Romanovsky, V. E. Long-Term Release of Carbon Dioxide from Arctic Tundra Ecosystems in Alaska. *Ecosystems* **20**, 960–974 (2017).

55. Pastorello, G. *et al.* The FLUXNET2015 dataset and the ONEFlux processing pipeline for eddy covariance data. *Sci. Data* **7**, 225 (2020).

56. Steffen, K., Box J., E. & Abdalati, W. *Greenland Climate Network: GC-Net*. *CRREL Special Report* 98-103 (CRREL, 1996).

57. CAVM Team. Circumpolar Arctic Vegetation Map. (1:7,500,000 scale). Conservation of Arctic Flora and Fauna (CAFF) Map No. 1. (2003).

58. Rossow, W. *et al.* International Satellite Cloud Climatology Project (ISCCP) Climate Data Record, H-Series. *NOAA National Centers for Environmental Information* (2017) doi:10.7289/v5qz281s.

59. Young, A. H., Knapp, K. R., Inamdar, A., Hankins, W. & Rossow, W. B. The International Satellite Cloud Climatology Project H-Series climate data record product. *Earth Syst. Sci. Data* **10**(1), 583-593 (2018). doi:10.5194/essd-10-583-2018.

60. Heginbottom, J., Brown, J., Ferrians, O. & Melnikov, E. S. Circum-Arctic Map of Permafrost and Ground-Ice Conditions, Version 2. *NSIDC* (2002) doi:10.7265/skbg-kf16.

61. Yurtsev, B. A. Floristic division of the Arctic. *J. Veg. Sci.* **5**(6), 765-776 (1994). doi:10.2307/3236191

62. Walker, D. A. *et al.* Circumpolar Arctic vegetation: a hierarchic review and roadmap toward an internationally consistent approach to survey, archive and classify tundra plot data. *Environ. Res. Lett.* **11**, 055005 (2016).

63. Raynolds, M. K., Comiso, J. C., Walker, D. A. & Verbyla, D. Relationship between satellite-derived land surface temperatures, arctic vegetation types, and NDVI. *Remote Sens. Env.* **112**(4), 1884-1894 (2008).
